# Individualizing Pharmacotherapy in Patients with Renal Impairment: The Validity of the Modification of Diet in Renal Disease Formula in Specific Patient Populations with a Glomerular Filtration Rate below 60 Ml/Min. A Systematic Review

**DOI:** 10.1371/journal.pone.0116403

**Published:** 2015-03-05

**Authors:** Willemijn L. Eppenga, Cornelis Kramers, Hieronymus J. Derijks, Michel Wensing, Jack F. M. Wetzels, Peter A. G. M. De Smet

**Affiliations:** 1 Radboud University Medical Center, Radboud Institute for Health Sciences, IQ Healthcare, Nijmegen, The Netherlands; 2 Radboud University Medical Center, Department of Pharmacology and Toxicology, Nijmegen, The Netherlands; 3 Department of Clinical Pharmacy, Canisius Wilhelmina Hospital, Nijmegen, The Netherlands; 4 Hospital Pharmacy ‘ZANOB’, ‘s-Hertogenbosch, The Netherlands; 5 Division of Pharmacoepidemiology and Pharmacotherapy, Utrecht Institute for Pharmaceutical Sciences (UIPS), Utrecht University, Utrecht, The Netherlands; 6 Radboud University Medical Center, Department of Nephrology, Nijmegen, The Netherlands; 7 Radboud University Medical Center, Department of Pharmacy, Nijmegen, The Netherlands; The University of Tokyo, JAPAN

## Abstract

**Background:**

The Modification of Diet in Renal Disease (MDRD) formula is widely used in clinical practice to assess the correct drug dose. This formula is based on serum creatinine levels which might be influenced by chronic diseases itself or the effects of the chronic diseases. We conducted a systematic review to determine the validity of the MDRD formula in specific patient populations with renal impairment: elderly, hospitalized and obese patients, patients with cardiovascular disease, cancer, chronic respiratory diseases, diabetes mellitus, liver cirrhosis and human immunodeficiency virus.

**Methods and Findings:**

We searched for articles in Pubmed published from January 1999 through January 2014. Selection criteria were (1) patients with a glomerular filtration rate (GFR) < 60 ml/min (/1.73m^2^), (2) MDRD formula compared with a gold standard and (3) statistical analysis focused on bias, precision and/or accuracy. Data extraction was done by the first author and checked by a second author. A bias of 20% or less, a precision of 30% or less and an accuracy expressed as P_30_% of 80% or higher were indicators of the validity of the MDRD formula. In total we included 27 studies. The number of patients included ranged from 8 to 1831. The gold standard and measurement method used varied across the studies. For none of the specific patient populations the studies provided sufficient evidence of validity of the MDRD formula regarding the three parameters. For patients with diabetes mellitus and liver cirrhosis, hospitalized patients and elderly with moderate to severe renal impairment we concluded that the MDRD formula is not valid. Limitations of the review are the lack of considering the method of measuring serum creatinine levels and the type of gold standard used.

**Conclusion:**

In several specific patient populations with renal impairment the use of the MDRD formula is not valid or has uncertain validity.

## Introduction

Chronic kidney disease (CKD) is a common condition and affects up to 13% of the population.[1] CKD is defined as a glomerular filtration rate (GFR) < 60 ml/min/1.73m^2^ or evidence of kidney damage (proteinuria, haematuria and/or abnormalities of the kidney) for at least 3 months regardless of underlying cause.[2,3] CKD is associated with adverse outcomes, such as kidney failure, cardiovascular diseases and death.[2,4,5] Since laboratories routinely report the estimated GFR (eGFR) if serum creatinine testing is ordered, the awareness of impaired renal function among physicians has increased in recent years.[6,7,8]

The eGFR is not only used to diagnose CKD or to monitor its course in patients with kidney disease, but also to guide decisions in pharmacotherapy. Potential uses of the eGFR in drug therapy are: (1) signal that treatment of CKD is warranted, (2) signal that a drug may be contraindicated, (3) signal that renal drug toxicity is developing, (4) signal that the risk of an adverse drug reaction or drug-drug interaction may be increased or (5) signal that a drug may be less effective. Approximately 20–30% of adverse drug reactions (ADRs) leading to hospital admission of elderly patients are related to impaired renal function.[9,10] This was mainly due to excessive doses of drugs and could have been avoided by close monitoring of renal function and adjustment of pharmacotherapy in terms of the prescribed agent(s) and/or the prescribed dosage(s).[9]

Drug dosing recommendations traditionally have used the Cockcroft and Gault (CG) formula to estimate creatinine clearance and therefore the ability of the kidney to excrete drugs.[6,11] This formula was developed in 249 adult men by using the mean 24-h urine creatinine excretion from two urine collections.[12,13]. The adjustment factor for women was based on a theoretical 15% lower muscle mass.[13,14] Approximately 15 years ago a new formula was developed that provided a more accurate estimation of GFR.[15,16] The original six variable Modification of Diet in Renal Disease (MDRD) formula, MDRD-6, was developed in a sample of 1070 ambulatory, predominantly white patients with CKD.[15] The six variables were serum creatinine concentration, age, sex, ethnicity, and serum urea nitrogen and albumin concentrations.[15] Several years later (in the year 2000), this formula was simplified to 4 variables (serum creatinine concentration, age, sex and ethnicity), MDRD-4.[16] The latter is now routinely used by many clinical laboratories worldwide.[17,18,19,20] Variability in the use of different creatinine assays among clinical laboratories led to the introduction of isotope dilution mass spectrometry (IDMS) calibration of the creatinine assays.[17,21,22] This led to a re-expression of the MDRD formulas. The MDRD formulas are presented in Box 1.

**Box 1. MDRD equations.**

MDRD-6 [15]

170 x S_cr_^−0.999^ x age^−0.176^ x BUN^−0.170^ x albumin^+0.318^x 1.180 (if black) x 0.762 (if female)

Re-expressed after IDMS calibration [21]

161.5 x S_cr_^−0.999^ x age^−0.176^ x BUN^−0.170^ x albumin^+0.318^x 1.180 (if black) x 0.762 (if female)

MDRD-4 [16]

186 x S_cr_^−1.154^ x age^−0.203^ x 1.212 (if black) x 0.742 (if female)

Re-expressed after IDMS calibration [21]

175 x S_cr_^−1.154^ x age^−0.203^ x 1.212 (if black) x 0.742 (if female)

S_cr_ = serum creatinine (mg/dL)

BUN = blood urea nitrogen (mg/dL)

Albumin (g/dL)

Although there is an ongoing debate on whether the MDRD formula can safely replace the CG formula in drug dosing [20,23], the MDRD formula is now widely used in clinical practice for drug dosing in various patient populations.[19,24,25] The aim of this article is to review systematically the validity and limitations of the MDRD formula in specific patient populations with a known glomerular filtration rate below 60 ml/min where adjustment of the pharmacotherapy usually should take place.

## Background

The MDRD formula is an estimation of the true GFR. The true GFR is the product of the filtration rate in single nephrons and the number of nephrons in both kidneys.[4] The ideal filtration marker to determine the true GFR is freely filtered across capillary walls, unhindered by its size, charge or binding to plasma proteins and neither secreted nor reabsorbed.[4] Inulin fulfils these criteria, but is not widely used for this purpose in clinical practice, because of the necessity for intravenous infusion and the difficult chemical assay required for inulin measurement.[26] The true GFR can also be measured using other markers such as ^51^chromium ethylenediaminetetraacetic acid (^51^Cr-EDTA), technetium-labelled diethylene-triamine-pentacetate (^99m^Tc-DTPA), iohexol and iothalamate.[27] However, these markers are also impractical for routine clinical use due to limited access to necessary diagnostic facilities and high costs.[3,28]

**Fig 1:**
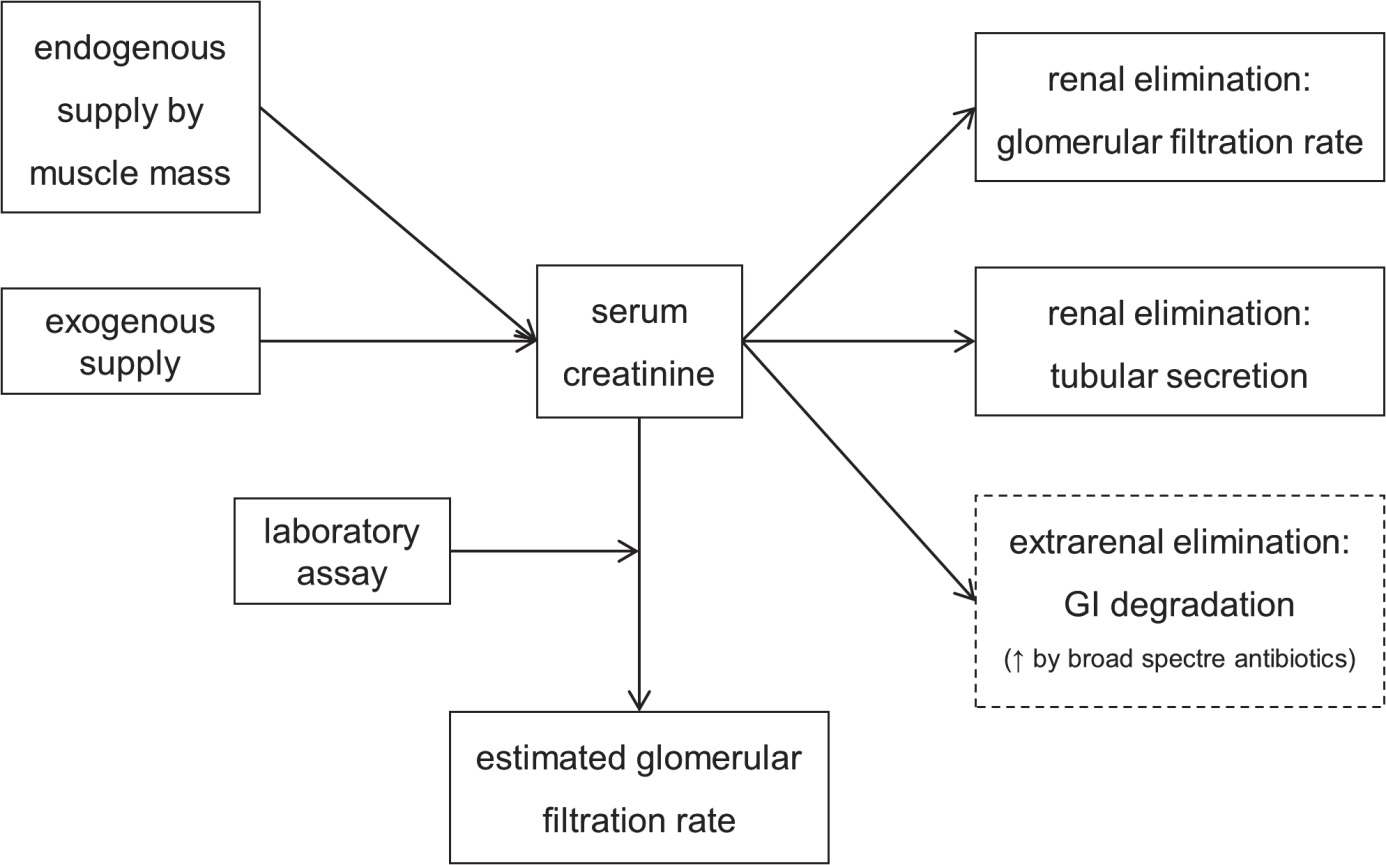
Determinants of serum creatinine level.

Creatinine is generally considered a good filtration marker to estimate the renal function in routine clinical practice. Serum creatinine level is a function of endogenous creatinine production, exogenous creatinine supply and renal elimination (glomerular filtration and tubular secretion).[29] There is a clear inverse correlation between serum creatinine levels and the true GFR. However there are several factors which may influence serum creatinine levels without affecting GFR itself, which potentially distort the interpretation of values for clinical use (see Fig. 1).[3,4,27,30,31]

### Endogenous creatinine production

Creatinine is formed primarily in skeletal muscles from creatine and creatine phosphate.[29] Muscle mass is the most important determinant of total body creatine content and therefore of the creatinine production.[26] Muscle mass is related to age, sex and race. [4,26,32] Although the MDRD formula corrects for age, sex and race, the formula assumes an average muscle mass. However, muscle mass can deviate across individuals in anabolic or catabolic conditions. Thus, muscle-waisting conditions, e.g. due to neuromuscular disease, chronic glucocorticoid therapy, hyperthyroidism, amputation or progressive muscular dystrophy are associated with decreased creatinine production, whereas exercise and body building are associated with increased creatinine generation. [4,26,32] In diseases with abnormally low muscle mass, serum creatinine levels will be relatively low, leading to an overestimation of the GFR, whereas the opposite will occur when the muscle mass is abnormally high.

In addition, the liver plays a major role in the biosynthesis of creatine. The rate of creatine formation and therefore the rate of creatinine production in the muscles is reduced in certain types of hepatic diseases.[33]

### Exogenous creatinine supply

Dietary intake of creatinine and creatine can be either unusually high (ingestion of cooked meat, creatine supplementation) or unusually low (vegetarian diet).[4,26] This may lead to underestimation and overestimation of the GFR, respectively.

### Laboratory assay of serum creatinine

Two assays are now mostly used in clinical practice to measure serum creatinine levels, namely the alkaline picrate assay (Jaffe) and the enzymatic assay. The Jaffe assay is known to have more interfering substances than the enzymatic method, which may result in a deviation of the true serum creatinine level up to 20%.[3,34] Substances which interfere with the Jaffe assay and may lead to an overestimation of serum creatinine levels include bilirubine, 5-aminolevulic acid, and high dose of lactulose. High doses of furosemide may lead to an underestimation of serum creatinine levels and cephalosporins may lead to both over- and underestimation of serum creatinine levels.[35,36,37,38,39] Substances which interfere with the enzymatic assay include dopamine, dobutamine, glucose and flucytosine.[26,35,36] These interferences may lead to an underestimation of serum creatinine levels and therefore an overestimation of the GFR, except for flucytosine. Flucytosine may overestimate serum creatinine levels with more than 100% and therefore underestimate the GFR.[36]

In addition to interferences of certain drugs in the creatinine assays, there are also differences in creatinine values between clinical laboratories due to differences in the creatinine assays and their calibration. Therefore a uniform creatinine measurement and a universal known calibration of the serum creatinine assays has led to the introduction of IDMS calibration.[18,40]

### Creatinine secretion

Creatinine is not only excreted by glomerular filtration, but also by the renal tubules. In addition, in patients with severe renal impairment a substantial fraction of the daily creatinine production is eliminated via extrarenal routes.[26] This might lead to relatively low serum creatinine levels, leading to an overestimation of the GFR. The creatinine secretion might also be altered in situations such as trauma, prolonged immobilization and hepatic disease.[26] The etiology of these changes is unknown. Some drugs, such as cimetidine, trimethoprim and fibrates (except gemfibrozil) also alter the secretion of creatinine due to inhibition of the tubular secretion.[4,36,41] As a result serum creatinine levels will increase, independent of the GFR, and the use of serum creatinine based formulas will thus cause an underestimation of GFR. Of note, blocking creatinine secretion ensures a better reflection of the true GFR with creatinine-based formulas.[42,43]

Serum creatinine levels thus provide only a rough guide to the true GFR.[26] While the dependence of serum creatinine on muscle mass is partially accounted for in the MDRD formula (age, sex and race), various other factors influencing both production and secretion of creatinine can be present. Awareness of these limitations of creatinine-based formulas which estimate GFR is therefore necessary. In addition, variability in eGFRs also includes underlying biological intraindividual and interindividual variation of serum creatinine values ranging from 6.3% to 17%.[12,22,44]

Of note, serum creatinine levels and creatinine based formulas should only be used in patients with stable renal function. In cases of rapidly changing GFR, the actual serum creatinine levels will not reflect the GFR, until steady-state has been reached.[45] A diagnosis of a low GFR must therefore rely on multiple measures of serum creatinine levels.[46]

## Methods

### Search strategy

We performed a systematic search in the Pubmed database for published studies about the validity of the MDRD formula in diverse patient populations. The search focused on publications between January 1999 (the introduction of the MDRD formula [15]) and January 2014. We searched for both the MDRD-4 and MDRD-6 formulas. The terms used for the overall search strategy have been listed in S1 File.

### Selection criteria

We focused on studies in patients with a measured GFR (mGFR) or estimated GFR (eGFR) < 60 ml/min(/1.73m^2^). Other selection criteria were (1) MDRD formula compared with a gold standard (defined as: ^99m^Tc-DTPA, inulin (including the analogue sinistrin[47]), ^51^Cr-EDTA, ^125^I-iothalamate and Iohexol) and (2) statistical analysis and reporting focused on bias, precision and/or accuracy (see Table 1 for definitions).

**Table 1:** Definitions outcome measurements.

Definition bias	Formula	Ref.
Median difference	md eGFR-mGFR	[67,120]
Median percentage difference*	md ((eGFR-mGFR)/mGFR) x 100%	[120]
Mean difference	1/n x Σ (eGFR-mGFR)	[135]
Mean percentage difference^‡^	1/n x Σ ((eGFR-mGFR)/mGFR) x 100%	[142]
**Precision**	**Formula**	**Ref**.
Inter quartile range (IQR) difference	IQR of (eGFR—mGFR)	[67]
IQR percentage difference*	IQR of ((eGFR-mGFR)/mGFR) x 100%	[67]
Limits of agreement (LOA)	Mean difference ± 1.96 SD	[135,136]
Standard deviation difference (SD)	σ of all the individual differences	[67,135]
**Accuracy**	**Formula**	**Ref**.
P_k_ ^§^	Percentage of estimates within k% of mGFR	[67]
Median absolute percentage error (mAPE)	md ((|eGFR—mGFR|)/mGFR) x 100%	[142]
Mean absolute percentage error (MAPE)	1/n x Σ ((|eGFR—mGFR|)/mGFR) x 100%	[142]

* Preferred definition because a relative scale provides a more relevant metric.

^‡^ In some articles the mean percentage difference was called the mean percentage error (MPE).

^§^ Preferred definition of accuracy. We limited our search to P_10_, P_20_, P_30_ and P_50_.

We excluded case reports, abstracts, and posters. Articles which reproduced data already published elsewhere were carefully reviewed. Only if newer data added information to our review, the article was included.

### Selection of patient population

In this review we will first discuss three more general patient groups, which were inadequately represented in the development of the MDRD formula, namely 1) elderly patients, 2) hospitalized patients and 3) obese patients.

The MDRD formula was developed in a relatively young patient population (mean age: 51 + 13 years) with CKD.[15,48] The mean body weight was 79.6 + 16.8 kg and the mean body surface area (BSA) was 1.91 + 0.23 m^2^.[15] The body mass index (BMI) calculated from the mean weight and BSA is approximately 28 kg/m^2^, so the population was not obese (> 30 kg/m^2^) as a whole. Creatinine production decreases as muscle mass decreases with age or due to immobility, which is common in hospitalized patients.[49] When this results in low serum creatinine levels creatinine-based formulas may overestimate the GFR.[50] In addition, elderly, malnourished, and immobilized patients are at special risk of having depressed GFR but normal serum creatinine levels, so normal serum creatinine concentration cannot exclude significant renal impairment.[51,52,53] Because of these considerations we found elderly patients (> 65 years), hospitalized patients and obese patients (> 30 kg/m^2^) of interest for further evaluation.

Second, we will discuss four common categories of chronic diseases, which are the leading cause of death in the developed world: 4) cardiovascular diseases (e.g. myocardial infarction, heart failure and stroke), 5) cancer, 6) chronic respiratory diseases (like chronic obstructed pulmonary disease (COPD) and asthma), and 7) diabetes mellitus.[54] These diseases are associated with the use of multiple drugs of which a substantial part is renally excreted.

The chronic diseases itself or the effects of the chronic diseases may alter serum creatinine levels without affecting GFR itself. In patients with chronic heart failure, a cardiovascular disease, the effective circulating volume is reduced, blood pressure is low and therefore renal perfusion pressure is reduced, leading to reduced filtration rate in viable nephrons and probably also to reduced excretion of creatinine. [55,56] The tubuli, however, are still capable of secreting creatinine actively.[55] In addition, the cornerstone in heart failure therapy are renin-angiotensin-aldosterone-system (RAAS) inhibitors, which also may reduce glomerular filtration pressure and therefore excretion of creatinine.[56] These different mechanisms may influence serum creatinine levels. In addition, patients with heart failure are often immobile and therefore at risk for having lower serum creatinine levels.

In cancer and COPD unknown mechanisms may influence creatinine levels without affecting GFR, but reduced muscle mass and malnourishment may also be present. This latter may result in low serum creatinine levels and therefore in overestimation of the GFR.

In diabetes mellitus, the choice of drugs or dosages is influenced by GFR.[57]

Finally, we searched for articles about 8) other chronic diseases in which reduced muscle mass (mainly due to immobility and malnutrition) can be present, which may render the MDRD formula less valid. Such diseases include neuromuscular diseases, rheumatoid arthritis, cystic fibrosis, human immunodeficiency virus (HIV) and liver diseases.[58,59,60,61,62] In certain liver diseases the production of serum creatinine is also reduced to approximately one half of the rate of patients with normal hepatic function.[58,60,63] Hyperbilirubinemia is also common among patients with liver diseases. Elevated serum bilirubine levels interfere with the Jaffe method to measure creatinine, which might lead to misleadingly low serum creatinine levels.[35,58,64]

### Extraction of studies

The data and outcomes reported in the selected articles were summarized, specifically focusing on the selection of the study population, age, mean mGFR, type of creatinine assay used, type of gold standard used, mean eGFR and the outcome measures as defined in Table 1. Data extraction was done by the first author (WE) and checked by a second author (MW). Description of the findings focused on:

- number of patients included;
- outcome measures;
- method of measuring true GFR.[27,65]

Ad (a) number of patients included: Preferably more than 100 patients should be included. [5,17] Studies with more than 100 patients included were considered of higher value in the interpretation than studies with less than 100 patients.

**Fig 2:**
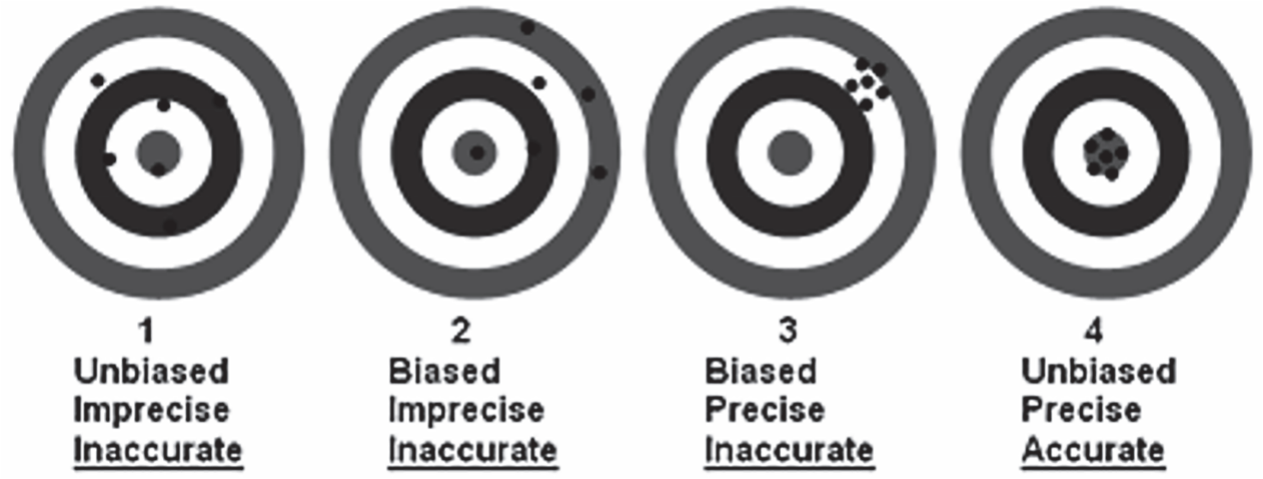
Precision and accuracy. Source: http://www.nrcan.gc.ca/minerals-metals/non-destructive-testing/application/2914,

Ad (b) outcome measures: One of our inclusion criteria concerned the presence of outcome measures: bias, precision and accuracy. Bias represents systematic error, precision represents random error and accuracy represents both (see Fig. 2).

In developing a model or formula, bias can to some extent be corrected by means of a correction factor. An example is the correction factor of 1.212 for Black-Americans in the MDRD formula.[15] Remaining bias refers to confounding factors for which corrections have not been included. It is not possible to completely correct for lack of precision (random error) in a model or formula. Precision is therefore an important indicator for the evaluation of the reliability of a clinical measure. Precision has been defined as the variance of the bias. The wider the variance of the bias, the higher the number that represents the precision, or in other words a wide imprecision. Accuracy is an validity indicator, which represents both precision and bias, thus systematic and random error. Accuracy is often expressed as a percentage of estimates within k% of mGFR (P_k%_). This outcome measure is probably most easily to interpret from a clinical perspective. For example, if P_30_ is 50%, it means that in half of the cases the eGFR falls within + 30% of the mGFR. Due to intraindividual and interindividual variation of serum creatinine levels and analytical variation of the measurement of serum creatinine levels and other influencing factors a P_30%_ of 100% will hardly be achievable.[5,12,22] In line with other authors we considered a bias of 20% or less, a precision of 30% or less and an accuracy expressed as P_30%_ of 80% or higher as indicator of sufficient validity.[14,66,67]

Ad (c) method of measuring true GFR: Several methods for true GFR measurement are available, including urinary clearance, plasma clearance or a combination of both from exogenous markers, such as ^99m^Tc-DTPA, ^51^Cr-EDTA, ^125^I-iothalamate and Iohexol.[68,69] With urinary clearance, two to four 20 to 30 minutes urine collections are obtained, after administration of the exogenous marker. Urinary clearance is computed as the urine concentration of the exogenous marker multiplied by the volume of the timed urine sample, and divided by the average plasma concentration during the same time period.[27] Plasma clearance is computed from the amount of the exogenous marker administered divided by the area under the curve of plasma concentration over time. The best estimate is a two-compartment model that requires blood sampling two to three time points until 60 minutes and one to three time points from 120 minutes forward.[27]

The clearance of inulin is determined by collecting three sets of 30 minutes urinary clearance periods during a continuous intravenous infusion with 1% of inulin. Blood samples should be collected at least three times at the midpoint of the urine collections. Bladder catheterization is necessary to assure complete urine collection.[27,70] Tests containing sinistrin, an analogue of inulin and also a fructan, is frequently used as a gold standard for the measurement of GFR and therefore included in our review.[47,71]

### Results and Interpretation

**Fig 3:**
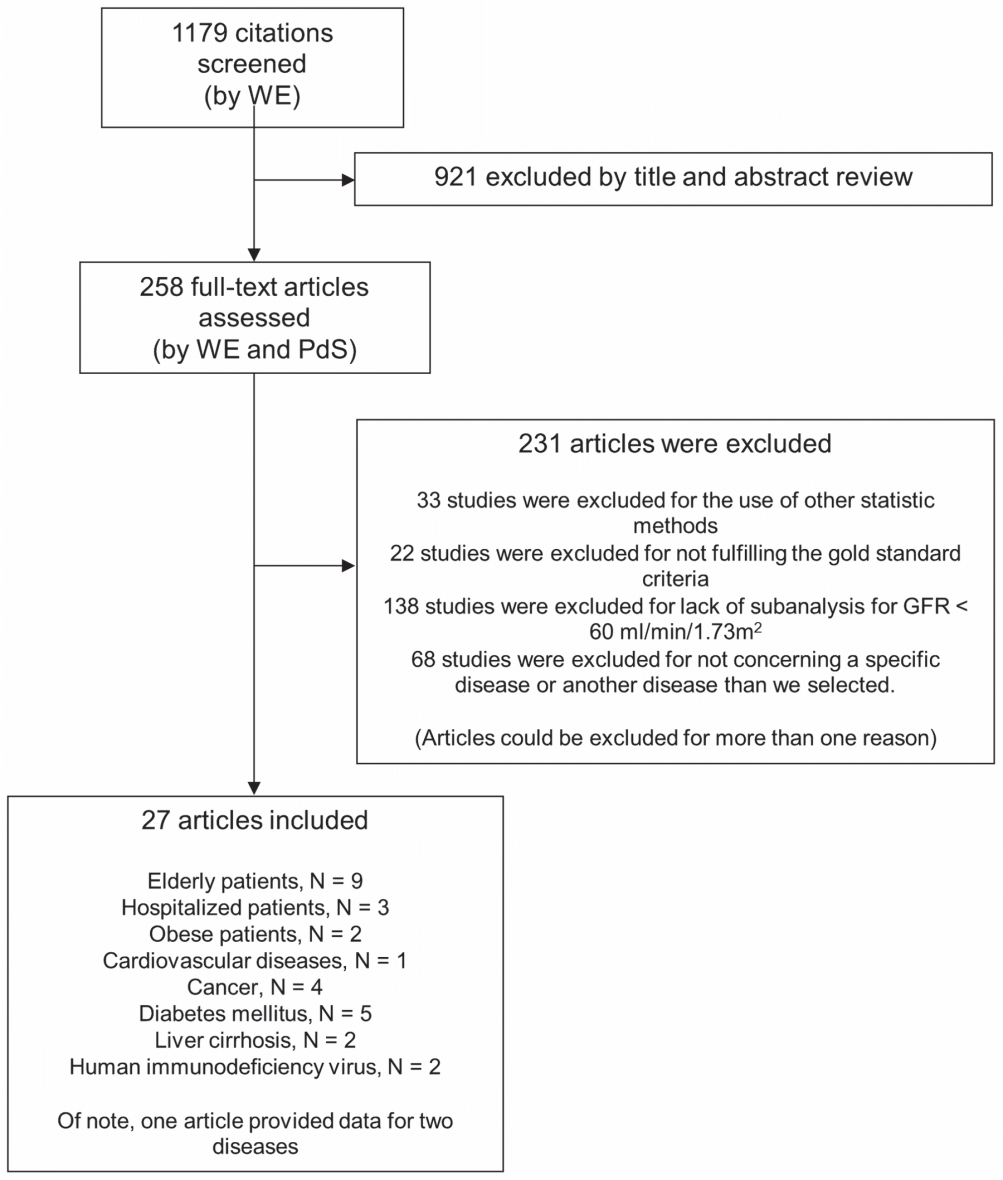
Flow of study selection.

We identified 1179 citations with the search terms listed in S1 File. In total we included 27 studies. The flow of study selection is reported in Fig. 3.

In Table 2 the most important information from the selected studies is summarized.

**Table 2:** Validity of the MDRD in specific patient populations.

Article	Study population	Mean(SD) Age(years)	Mean (SD) mGFR	Gold standard	Creatinine measurement	Mean (SD) eGFR(MDRD)	Bias	Precision	Accuracy
***Elderly patients***									
Lopes et al., 2013, Brasil [143]	N = 56; subanalysis: mGFR < 60 ml/min/1.73m^2^; inclusion criteria: age > 80 years, clinically stable, independent in activities; exclusion criteria: to be institutionalized, unable or willing to give consent, acute infection, moderate or severe cognitive impairment, heart failure, cirrhosis, previously received dialysis, unstable COPD, previous immunosuppressive therapy within 6 months, previous chemotherapy for cancer, known HIV infection, and previously reported allergic reaction to iodine.	-*	-*	Iohexol; Blood samples were withdrawn 2, 3, 4 and 5 h after infusion	Jaffe	-*	*Re-expressed MDRD-4*: mean: 5.9 ml/min/1.73m^2^	*Re-expressed MDRD-4*: SD: 14.1 ml/min/1.73m^2^	*Re-expressed MDRD-4*: P_30_: 64.3%
Evans et al., 2013, Sweden [80]	N = 1831; subanalysis: age > 65 years; inclusion criteria: Patients with an iohexol measurement < 30 ml/min/1.73m^2^, a registered plasma creatinine on the same date, between 1999 and 2010. exclusion criteria: renal transplants, dialysis, patients from the Lund-Malmo region.	-*	Median (IQR): 15 (12–20) ml/min/1.73m^2^	Iohexol; Blood samples were withdrawn at baseline and after 6–8h or 48h after injection, for patients with eGFR between 15–30 ml/min/1.73m^2^ and < 15 ml/min/1.73m^2^, respectively.	Enzymatic or Jaffe	-*	-*	-*	*Re-expressed MDRD-4*: P_30_: 64.6%
Koppe et al., 2013, France [71]	N = 53, KDIGO CKD stage 3A, N = 68, KDIGO CKD stage 3B, N = 66, KDIGO CKD stage 4–5; inclusion criteria: age > 70 years, white (Caucasion), underwent inulin clearance for suspected or established renal dysfunction.	-*	-*	Sinistrin; Loading dose followed by continuous infusion. Urine and plasma collection. (time and numbers not described)	Enzymatic	-*	*Re-expressed MDRD-4* ^*≠*^: Stage 3A: median: 2 ml/min/1.73m^2^; Stage 3B: median: 6.7 ml/min/1.73m^2^; Stage 4–5: median: 5.95 ml/min/1.73m^2^	*Re-expressed MDRD-4* ^*≠*^: Stage 3A: SD: 13.53 ml/min/1.73m^2^; Stage 3B: SD: 11.69 ml/min/1.73m^2^; Stage 4–5: SD: 8.6 ml/min/1.73m^2^	*Re-expressed MDRD-4* ^*≠*^: Stage 3A: P_10_: 41.51%, P_30_: 84.91%; Stage 3B: P_10_: 28.99%, P_30_: 73.91%; Stage 4–5: P_10_: 19.7%, P_30_: 59.09%
Drenth-van Maanen et al., 2013, Netherlands [47]	N = 16; inclusion criteria: Patients with GFR(MDRD) < 60 ml/min/1.73m^2^, age > 70 years, stable medical condition and cognitively able to give informed consent at acute care and outpatient geriatric ward.	82 range: 71–87	39.6 (14.9)^$^ ml/min/1.73m^2^	Sinistrin; Bolus injection, blood samples were withdrawn at 10, 20, 30, 60, 90, 120, 240 and 480 min after infusion.	Jaffe	*MDRD-4*: 48.6 (13.8)^$^: ml/min/1.73m^2^	*MDRD-4*: mean (%): 29.1	*MDRD-4*: LOA: -16–34 ml/min/1.73m^2^	*MDRD-4*: P_30_ = 62.5%
Kilbride et al. 2012, United Kingdom [77]	N = 234; subanalysis: mGFR < 60 ml/min/1.73m^2^; inclusion: > 74 years; exclusion: iodinated contrast media allergy, active malignancy, life expectancy less than 3 months, cognitive impairment, recent episode (within 3 months) of AKI, dialysis.	-*	-*	Iohexol; Bolus injection, blood samples were withdrawn at 120, 180 and 240 minutes.	Enzymatic	-*	*Re-expressed MDRD-4*: median: 2.0 ml/min/1.73m^2^	*Re-expressed MDRD-4*: IQR: 11.4 ml/min/1.73m^2^	*Re-expressed MDRD-4*: P_30_: 78%
Bevc et al., 2011, Slovenia [76]	N = 266; subanalysis: mGFR < 60 ml/min/1.73m^2^; inclusion criteria: age > 65 years, Caucasian.	-*	-*	^51^CrEDTA; After single injection blood samples were withdrawn at 120, 180 and 240 minutes	Jaffe	-*	*MDRD-4*: mean: -20.2 ml/min/1.73m^2^	*MDRD-4*: SD: 14.9 ml/min/1.73m^2^	*MDRD-4*: 30–59 ml/min/1.73m^2^: P_30_: 77.9%; 15–29 ml/min/1.73m^2^: P_30_: 56.6%; <15 ml/min/1.73m^2^: P_30_: 55.2%
Stevens et al., 2007, United States [79]	N = 580; subanalysis: age > 65 years and eGFR < 60 ml/min/1.73m^2^; the results have been compiled from data from different studies.	-*	-*	Iothalamate; Drawing of samples was not described	-*	-*	*MDRD-4*: median: -1.2%	-*	*MDRD-4*: P_30_: 82%
Fontsere et al., 2006, Spain [144]	N = 43; subanalysis: age > 65 years; inclusion criteria: Caucasian adult patients with CKD stages 4–5.	-*	22.9 + 6.8 ml/min/1.73m^2^	^51^CrEDTA; Drawing of samples was not described	Jaffe	-*	*MDRD-4*: mean: -4.1 ml/min/1.73m^2^	-*	-*
Froissart et al., 2005, France [78]	N = -*; subanalysis: age > 65 years and mGFR < 60 ml/min/1.73m^2^; exclusion criteria: renal transplant patients and age < 18 years, black patients.	-*	-*	^51^CrEDTA; Collection of urine 1 hour after injection and then 5 consecutive 30-min clearances. Blood was drawn at midpoint of each clearance period up to 300 min after injection.	Jaffe	-*	*MDRD-4*: male: mean (%): 5.6; female: mean(%): 7.6	*MDRD-4*: male: SD(%): 31.4; female: SD(%): 34.1	-*
***Hospitalized patients***									
Frank et al., 2012, Switzerland [145]	N = 69; inclusion criteria: Caucasian patients, age >70 years, with CKD stage III-IV according to KDOQI guidelines of the internal medicine ward. Stable weight for 4 days; exclusion criteria: unstable renal function in the last two weeks.	Median (IQR): 80 (73–83)	Median (IQR): 30.9 (22.0–43.3) ml/min	Inulin; Blood samples were withdrawn at baseline, 90, 180, 270 and 360 minutes.	Jaffe	*Re-expressed MDRD-4*: median: 47.9 ml/min	*Re-expressed MDRD-4*: median: 16.3 ml/min	*Re-expressed MDRD-4*: IQR: 6.4–27.5 ml/min	-*
Poggio et al., 2005, California [85]	N = 107; inclusion criteria: patients who had mGFR performed with varying degrees of kidney dysfunction; exclusion criteria: incomplete data, dialysis, serum creatinine level < 0.3mg/dl (<27umol/l), unstable renal function.	65 + 15	17.1 + 17.9 ml/min/1.73m^2^	Iothalamate; Bolus injection, blood samples were withdrawn at 5, 10, 15, 300, 330 and 360 minutes. In case of expected GFR <30, age >65, or creatinine level > 2.5mg.dl, a sample at 24 hours was also collected.	Jaffe	*MDRD-4*: mean (SD): 23.9 + 16.3 ml/min/1.73m^2^; *MDRD-6*: mean (SD): 22.5 + 17.4 ml/min/1.73m^2^	*MDRD-4*: median(%): 53; *MDRD-6*: median(%): 46	-*	*MDRD-4*: MAPE: 53%, P_30_: 31%, P_50_: 49%; *MDRD-6*: MAPE: 47%, P_30_: 36%, P_50_: 55%
Schuck et al., 2005, Czech Republic [86]	N = 79; Nephrology Department; inclusion criteria: GFR < 50 ml/min/1.73m^2^; exclusion criteria: cachexia	Range: 20–65	19.1 + 10.1 ml/min/1.73m^2^	Inulin; After equilibrium phase (60 min) Urine collection during 60–90 min by spontaneous urination	Jaffe	*MDRD-6*: 22.1 + 8.3 ml/min/1.73m^2^	*MDRD-6*: mean: 3.26 ml/min/1.73m^2^	*MDRD-6*: LOA: -5.7–12.2 ml/min/1.73m^2^	-*
***Obese patients***									
Bouquegneau., 2013, Belgium [89]	N = 207; subanalysis: mGFR < 60 ml/min/1.73m^2^; inclusion criteria: patients were > 18 years and BMI > 30 kg/m^2^; exclusion criteria: patients treated with steroids, cimetidine or trimethoprim.	-*	36 + 13 ml/min/1.73m^2^	^51^Cr-EDTA; After single injection blood samples were drawn at 120 and 240 min.	Jaffe	*Re-expressed MDRD-4*: 36 + 17 ml/min/1.73m^2^	*Re-expressed MDRD-4*: mean (%): -0.8	*Re-expressed MDRD-4*: SD (%): 32	*Re-expressed MDRD-4*: P_30_: 80%
Stevens et al., 2007, United States [79]	N = 1039; subanalysis: BMI > 30 kg/m^2^ and eGFR < 60 ml/min/1.73m^2^; the results have been compiled from data from different studies.	-*	-*	Iothalamate; Drawing of samples was not described	-*	-*	*MDRD-4*: median(%): 4.1	-*	*MDRD-4*: P_30_: 82%
***Cardiovascular diseases***									
Valente et al., 2014, Netherlands [146]	N = 40; subanalysis: mGFR < 60 ml/min/1.73m^2^; inclusion criteria: age > 18 years, LVEF < 0.45, clinically stable, use of renin angiotensin system inhibitors; exclusion criteria: myocardial infarction within the last 3 months, cardiac surgery or angioplasty within the last 3 months or scheduled, unstable angina pectoris, primary renal disease, prior organ transplantation, chronic use of renal function-compromising medication.	-*	-*	^125^I-iothalamate; Constant infusion. 2-hour stabilization period.	Jaffe	-*	*Re-expressed MDRD-4*: mean: -2 ml/min/1.73m^2^	*Re-expressed MDRD-4*: SD: 9 ml/min/1.73m^2^	-*
***Cancer***									
Craig et al., 2012, United Kingdom [147]	N = -*; subanalysis: 30 < mGFR < 59 ml/min/1.73m^2^, mGFR < 30 ml/min/1.73m^2^; inclusion criteria: patient treated with chemotherapy following their mGFR, serum creatinine measured within 7 days of mGFR, serum creatinine > 60 umol/l, age > 20 years; exclusion criteria: patients with missing information, serum creatinine < 60 umol/l	-*	-*	^51^CrEDTA; After single injection blood samples were drawn at 120 and 240 minutes.	Jaffe	-*	*Re-expressed MDRD-4*: 30 < mGFR < 59: mean: 15.7 ml/min/1.73m^2^; mGFR <30: mean: 7.0 ml/min/1.73m^2^	-*	-*
Ainsworth et al., 2012, United Kingdom [106]	N = 45; patients who had mGFR < 50 ml/min. at the Department of Nuclear Medicine.	-*	-*	^51^Cr-EDTA; Single-sample method until 2005, thereafter three-sample method.	Jaffe	-*	*MDRD-4*: median(%): 17.5	-*	*MDRD-4*: median APE(%): 20.5
Bolke et al., 2011, Germany [104]	N = 8; subanalysis: mGFR < 60 ml/min/1.73m^2^; 8 patients with head and neck cancer presenting for combined radiochemotherapy and with known chronic renal insufficiency stage 3–5; exclusion: high dose steroid treatment.	-*	46.8 + 7.9^$^ ml/min/1.73m^2^	^51^Cr-EDTA; Bolus injection, four blood samples between 120 and 300 minutes after injection.	Enzymatic	*Re-expressed MDRD-4*: 55.2 + 13.2^$^ ml/min/1.73m^2^	*Re-expressed MDRD-4*: median(%): 18.4^$^	*Re-expressed MDRD-4*: SD(%): 19.1^$^	*Re-expressed MDRD-4*: P_30_: 75%^$^, P_50_: 100%^$^
Faluyi et al., 2011, United Kingdom [105]	N = 62; patients with mGFR < 60 ml/min with stable renal function at a Cancer Centre; exclusion: unstable renal function.	68.3 + 11.2	-*	^99m^Tc-DTPA; Bolus injection, blood samples withdrawn after 2 and 5 hours.	Enzymatic	-*	-*	-*	*Re-expressed MDRD-4*: P_10_: 40.3%, P_30_: 80.6%
***Diabetes mellitus***									
Iliadis et al., 2011, Greece [57]	N = 145; consecutive type 2 diabetic outpatients with mGFR between 30–59 ml/min/1.73m^2^	71 + 9	48.1 + 8.1 ml/min/1.73m^2^	^51^Cr-EDTA; Bolus injection, blood samples withdrawn after 2 and 4 hours.	Jaffe	*Re-expressed MDRD-4*: 56.0 + 13.0 ml/min/1.73m^2^	*Re-expressed MDRD-4*: mean: 7.5 ml/min/1.73m^2^	*Re-expressed MDRD-4*: SD: 9.5 ml/min/1.73m^2^	*Re-expressed MDRD-4*: P_10_: 26.3%, P_30_: 69.3%
Rognant et al., 2011, France [148]	N = 149; nondialyzed diabetic adult patients with mGFR < 60ml/min/1.73m^2^	-*	36.4 + 13 ml/min/1.73m^2^	Inulin; Continuous infusion, both blood and urine samples were withdrawn.	Jaffe	-*	-*	-*	*MDRD-4*: P_10_: 36.2%, P_30_: 75.3%
Fontsere et al., 2008, Spain [149]	N = 36; subanalysis: 15 < mGFR < 59 ml/min/1.73m^2^; Caucasian type 2 diabetic patients.	64 + 8.0	31.2 + 10.8 ml/min/1.73m^2^	^125^I-iothalamate; Drawing of samples was not described	Jaffe	-*	*MDRD-4*: -5.3 ml/min/1.73m^2^	-*	-*
Rigalleau, 2007, France [150]	N = 89; inclusion criteria: diabetes and an eGFR < 60 ml/min/1.73m^2^; exclusion criteria: renal replacement therapy	Normoalbuminuric; 68 + 9; Albuminuric: 64 + 12	45.6 + 29.7 ml/min/1.73m^2^	^51^Cr-EDTA; Bolus injection, four blood samples were drawn at 75, 105, 135 and 165 minutes and urine samples were collected at 90, 120, 150 and 180 minutes.	Jaffe	41.3 + 13.1 ml/min/1.73m^2^	-*	-*	*MDRD-*? *(not mentioned)*: Normoalbuminuric: P_10_:26%, P_30_: 73%, P_50_: 86%; Albuminuric: P_10_:24%, P_30_: 60%, P_50_: 79%
Rigalleau, 2005, France [110]	N = 87; diabetic patients with mGFR< 60 ml/min/1.73m^2^; exclusion: nephrotic proteinuria (>3g/24h), edema and dialysis.	-*	33.7 + 14.7 ml/min/1.73m^2^	^51^Cr-EDTA; Bolus injection, four blood samples were drawn at 75, 105, 135 and 165 minutes and urine samples were collected at 90, 120, 150 and 180 minutes.	Jaffe	*MDRD-4*: 38.4 + 14.0 ml/min/1.73m^2^	*MDRD-4*: mean: 4.7 ml/min/1.73m^2^	*MDRD-4*: 2SD: 20.6 ml/min/1.73m^2^	-*
***Liver cirrhosis***									
Mindikoglu et al., 2014, United States [115]	N = 21; subanalysis: mGFR < 60 ml/min/1.73m^2^; inclusion criteria: cirrhosis, age > 18 years; exclusion criteria: pregnancy or breast-feeding, iothalamate or iodine allergy, not treated hepatocellular carcinoma, hyperthyroidism, inability to provide informed consent or to collect or void urine, dialysis or eGFR < 15 ml/min/1.73m^2^, treatment with NSAIDs, ACE-inhibitors 1 week prior. Onset or change in diuretics 1 week prior. Acute infection, exacerbation of encephalopathy, gastrointestinal bleeding, kidney injury 1 week prior, acute cardiovascular or cerebrovascular event 3 weeks prior, and cognitive impairment.	-*	-*	Iothalamate; Blood samples were drawn at baseline, 5, 15, 30, 45, 60, 120, 240 and 360 minutes after iothalamate administration	-	-^*^	*Re-expressed MDRD-6* ^*≠*^: mean: -10.4 ml/min/1.73m^2^	*Re-expressed MDRD-6* ^*≠*^: SD: 13.65 ml/min/1.73m^2^	*Re-expressed MDRD-6* ^*≠*^: P_20_: 52.38%, P_30_: 61.90%
Rognant et al., 2010, France [114]	N = 45; consecutive candidates for liver transplantation with decompensated alcoholic cirrhosis with mGFR < 60 ml/min/1.73m^2^.	-*	-*	Inulin; Continuous infusion (2 to 2.5 hours), collection of three to four urine samples and a blood sample midway through each collection period.	^-^	-*	*MDRD-4*: mean: 19 ml/min/1.73m^2^	*MDRD-4*: SD: 25 ml/min/1.73m^2^	*MDRD-4*: P_10_: 11%, P_30_: 40%
***Human immunodeficiency virus***									
Gagneux et al., 2013, France [126]	N = 18; subanalysis: mGFR < 60 ml/min/1.73m^2^; inclusion criteria: age > 18 years, conformed HIV status; exclusion criteria: pregnancy, history of allergy, thyroid dysfunction, recent acute kidney injury, and treatment by metformin, steroids, trimethoprim, or cimetidine.	-	-	Iohexol; Bolus injection, blood samples withdrawn after 120 and 240 minutes.	Enzymatic	-	*Re-expressed MDRD-4*: mean(%): 61	*Re-expressed MDRD-4*: SD(%): 58	*Re-expressed MDRD-4*: P_30_: 22%
Inker et al., 2012, United States [127]	N = 27; subanalysis: eGFR < 60 ml/min/1.73m^2^; inclusion: age > 18 years, stable on antiretroviral therapy for at least three months, confirmed HIV status, HIV viral load and CD4 count within 6 months of recruitment; exclusion: pregnancy, allergy or contraindication for iohexol or iodine, recent acute kidney injury, cognitive or physical impairments, use of cimetidine.	-*	-*	Iohexol; Bolus injection, blood samples withdrawn after 10, 30, 120 and 240 minutes; for participants with serum creatinine > 1.5 mg/dl, a sample at 360 minutes was drawn.	-	-*	*Re-expressed MDRD-4*: median: -11.9 ml/min/1.73m^2^	*Re-expressed MDRD-4*: IQR: 19.4 ml/min/1.73m^2^	*Re-expressed MDRD-4*: P_30_: 66.7%

* Not all parameters were reported in the included articles. Especially when it came to subanalysis of patients with an eGFR < 60 ml/min.1.73m^2^.

^$^ When individual data were available we calculated missing parameters ourselves.

^*≠*^ The MDRD-formula used was not reported. Given the time at which the study was conducted, we assume that the re-expressed MDRD-formula was used.

The PRISMA checklist is provided as S1 PRISMA Checklist.

### Elderly patients

#### Background

CKD is common in elderly and is associated with a high risk of cardiovascular complications.[66,69] Early recognition, intervention and management of patients with CKD by physicians has been shown to slow progression of disease and decrease complications.[72] Accurate estimate of GFR is important to detect elderly patients at risk for progressive CKD, but also in order to guide drug therapy of potentially nephrotoxic drugs, or to adapt therapy with renally excreted drugs which is often desirable in elderly patients on polypharmacy.[73,74,75]

#### Summary of the selected articles

We included nine studies, four of which were conducted in 2013. The definition of elderly or older patients ranged from 65 to 80 years or older. The mean bias reported ranged from an underestimation of 20 ml/min/1.73m^2^ in the study of Bevc et al. to an overestimation of 29% in the study of Drenth-van Maanen et al.[47,76] Interpretation of precision is more difficult. The IQR of 11 ml/min/1.73m^2^ reported by Kilbride et al. is reasonable when considering persons with higher ranges of mGFR (around 60 ml/min/1.73m^2^), but would be less acceptable in the lower ranges of the mGFR. The accuracy reported as P_30_ was almost 80% which implies that the variance of the bias, the precision, is too large over the total range from 7 to 60 ml/min/1.73m^2^.[77] Although, four studies reported a bias within our 20% criteria[71,77,78,79], only two studies reported an accuracy above 80%, namely Stevens et al. and the subanalysis of the KDIGO CKD stage 3A in the study of Koppe et al.[71,79] In five studies (56%) the number of patients included exceeded 100.[71,76,77,79,80] Six out of nine studies described the method for measuring GFR adequately. The gold standards were reasonably performed, but only the studies of Drenth- van Maanen et al. and Froissart et al. met the number of blood samples taken over time as described above.[47,78]

#### Interpretation and conclusion

The nine included studies were performed reasonably. There was only one study[79] in which the MDRD formula appeared to be valid. Earlier Pottelbergh et al. conducted a systematic review with broader selection criteria and reported both over- and underestimation of the mGFR.[69] They concluded that there is no accurate creatinine-based formula to evaluate renal function in elderly patients.[69] With the more recently published studies presented here, we can confirm the conclusion that the creatinine-based MDRD formula is not valid in elderly patients.

### Hospitalized patients

#### Background

Reduced GFR is one of the most important complications in critically ill patients and is associated with increased morbidity and mortality in the intensive care unit (ICU) population.[81,82] In addition, acute renal failure (ARF) is also associated with high mortality.[83] Early detection of renal dysfunction and subsequent adequate treatment is therefore necessary in the hospital care setting.[84] Estimation of the GFR is also necessary for appropriate treatment of critically ill and other hospitalized patients with renally excreted drugs.[85]

#### Summary of the selected articles

In Table 2 three studies are presented, which fulfilled our inclusion criteria. All three studies reported an overestimation of the true GFR, ranging from a mean bias of 3.26 ml/min/1.73m^2^ in the study of Schuck et al to a median relative bias of 53% in the study of Poggio et al.[85,86] In the study of Poggio et al. the MDRD-6 formula seemed to perform slightly better than the MDRD-4 formula with a median bias of 46%.[85] Although the bias in the study of Schuck et al. seems low, the precision exceeded our criteria of 30%.[86] Only the study of Poggio et al reported accuracy, which appeared to be inadequate. P_30_ was 36% for the MDRD-6 formula and 31% for the MDRD-4 formula.[85] The measurement of the GFR with a gold standard was performed reasonably, although the spontaneous urination instead of catheterization in the study of Schuck et al. is questionable.[86] The number of patients only exceeded 100 in the study of Poggio et al.[85]

#### Interpretation and conclusion

The MDRD formula is invalid in hospitalized patients on the internal medicine and nephrology ward. In the study of Poggio et al. selection bias was introduced, because the selection of patients was based on an individual nephrologist’s perception of laboratory values not reflecting actual GFR.[85] In the other two studies the study was conducted only on the internal medicine and nephrology ward. The study population may therefore not reflect the average hospitalized patient population. In conclusion, the eGFR may largely overestimate true GFR in hospitalized patients, but the impact of this effect in different populations of hospitalized patients is still insufficiently known.

### Obese patients

#### Background

Obesity is a well-recognized global health problem.[87] Obesity is associated with cardiovascular complications, type 2 diabetes mellitus, osteoarthritis, major depression and some cancers.[7,87] Obesity itself and its combination with these chronic diseases predispose individuals to develop CKD.[7,88] Considering the increasing number of obese patients the accuracy of the MDRD formula in obesity is of increasing importance.

#### Summary of the selected articles

Two articles met our selection criteria. Both studies had a study population exceeding 100 patients. One study was compiled with data from different studies, so the measurement of the GFR was not described.[79] In the study of Bouquegneau et al. the measurement of the GFR was not adequately performed.[89] In both studies the true GFR and eGFR were normalized to ml/min/1.73m^2^. The bias was for both studies within our 20% criteria. The precision reported in the study of Bouquegnea et al. was slightly higher than 30%, but the accuracy was sufficient, P_30_ is 80%.[89] Stevens et al. reported also a sufficient accuracy, P_30_ of 82%.[79]

#### Interpretation and conclusion

We found two studies, which conducted a subanalysis about the validity of the MDRD-4 formula in obese patients (BMI > 30 kg/m^2^). The performance of the MDRD-4 formula seemed valid, but the measurement of the gold standard could have been performed better. In conclusion, we were not able to draw conclusions about the validity of the MDRD formula in obese patients.

### Cardiovascular diseases

#### Background

Numerous studies about the prognostic value of the eGFR(MDRD) in cardiovascular diseases for clinical outcomes, such as mortality, have been published.[56,90,91,92,93,94] In patients with end-stage heart failure, irreversibly impaired renal function precludes eligibility for heart transplantation.[95] In addition, patients with cardiovascular diseases are at risk for polypharmacy and the use of drugs that require dosage adjustment in renal impairment.[96,97] Thus an accurate method to estimate GFR is essential.[98]

#### Summary of the selected articles

One article met our selection criteria. The mean bias of −2 ml/min/1.73m^2^ with a precision of 9 ml/min/1.73m^2^ are within our criteria of 20% and 30%, respectively. However, the number of patients included was low. The measurement of the true GFR was not performed with a common method, a continuous infusion of ^125^I-iothalamate instead of a bolus injection.

#### Interpretation and conclusion

In conclusion, we were not able to draw conclusions about the validity of the MDRD formula in patients with heart failure (or other cardiovascular diseases).

### Cancer

#### Background

Both cancer and its drug therapies can lead to renal impairment.[99] Renal impairment in patients with cancer is highly prevalent and has major clinical implications.[99,100] In the Belgian Renal Insufficiency and Anticancer Medication (BIRMA) study, the prevalence of renal impairment (eGFR < 90 ml/min/1.73m^2^) in patients with a range of cancer diagnosis was 64%.[101] 80% of the patients treated for cancer received at least one nephrotoxic drug and/or drugs for which dosage had to be adjusted in renal impairment.[101] In our ageing societies oncologists are likely to be faced with increasing numbers of patients with both cancer and renal impairment.[102,103]

#### Summary of the selected articles

Four studies were selected and are presented in Table 2. None of these studies reported precision. Although the bias reported in the studies of Ainsworth et al. and Bolke et al. were within 20%, only the study of Faluyi et al. reported an accuracy >80% expressed as P_30_.[104,105,106] The low accuracy in the other studies implies a wide imprecision. The number of patients included in all three studies together exceeded 100. The measurement of GFR with a gold standard was not adequately performed in any of the studies. The best performed measurement of the GFR was in the study of Bolke et al. where 4 blood samples were withdrawn over a time period of 5 hours.[104]

#### Interpretation and conclusion

Precision was not reported, the numbers of patients in the selected separate studies were low and only the measurement of the GFR in the study of Bolke et al. seemed robust. Yet, these studies suggest that the eGFR calculated with the MDRD formula in cancer patients with moderate to severe renal impairment may be substantially different from the mGFR for a substantial number of patients. There is no evidence that the use of the MDRD formula in drug dosing in patients with cancer and renal impairment is valid.

### Chronic respiratory diseases

#### Background

The most frequent chronic respiratory disease is chronic obstructive pulmonary disease [107], which is associated with several comorbidities, such as hypertension, heart failure and diabetes.[107] Renal impairment is a significant risk factor for cardiovascular diseases for which COPD patients are at risk.[108] In addition, polypharmacy is frequent in patients with COPD.[108] An accurate estimation of the GFR seems therefore important.

#### Summary of the included articles

No articles met our selection criteria.

#### Interpretation and conclusion

When searching for articles about the validity of the MDRD formula in patients with COPD, we found some recently published articles on the prevalence of renal impairment in patients with COPD, which discussed the advantages of using creatinine-based formulas for estimating GFR rather than serum creatinine levels.[107,108] The prevalence of undiagnosed renal impairment (eGFR< 60 ml/min and normal serum creatinine levels) varied between 7 and 22%, and was higher in patients with cachexia and older age (>64 years).[107,108] This implies that the issue of the validity of the MDRD formula in COPD patients is still far from settled. In conclusion, we were not able to draw conclusions about the validity of the MDRD formula in patients with chronic respiratory diseases and moderate to severe renal impairment.

### Diabetes mellitus

#### Background

Diabetes mellitus is the leading cause of CKD.[109] Diabetic nephropathy affects around one-third of patients with diabetes and is the primary cause of end-stage renal disease worldwide.[57,109] Moreover, diabetic patients, especially those with impaired renal function, are at increased risk of cardiovascular events.[57,110] There is strong evidence that early detection of diabetic nephropathy leading to timely intervention improves long-term outcome.[111]

#### Summary of the selected articles

Several studies have been published about the validity of the MDRD-4 formula in patients with diabetes mellitus. We included five studies. The bias reported in three out of five studies were within 20%. The precision (only reported by Iliadis et al. and Rigalleau et al.) was in the same range, namely a SD of approximately 10 ml/min/1.73m^2^, and within our criterion of 30%.[57,110] Despite the fact that both bias and precision reported met our criteria, the accuracy was not sufficient, which implies a relatively wide imprecision throughout the mGFR range.

In two out of five studies the included number of patients exceeded 100. The findings of two other studies which included nearly 90 patients were in the same range. With respect to the method for measuring GFR, the number of blood samples withdrawn in the study of Iliadis et al. (two blood samples) was probably marginal.[57] In the other four studies the method for GFR measurement was sufficient.

#### Interpretation and conclusion

In four studies the MDRD-4 formula overestimated the mGFR. The overestimation of the GFR might lead to higher drug doses than necessary or to a late discontinuation of, for example, metformin use and therefore to a greater risk of adverse drug events. Although bias pointed in the same direction in four studies, the precision (= random error), which was not adequately reported, was probably too wide, which led to low accuracy. In conclusion, the MDRD formula is not valid in patients with diabetes mellitus and renal impairment.

### Other chronic diseases

From various chronic diseases of interest, we decided to present the diseases of which we could include at least two studies. These diseases were liver cirrhosis and HIV-infection.

### Liver cirrhosis

#### Background

Renal dysfunction often accompanies later stages of chronic liver diseases and is strongly associated with increased mortality in both acute liver failure and liver cirrhosis.[58,112] Pretransplant serum creatinine level is a predictor of posttransplant mortality and posttransplant renal function. [63,113] Other risk factors to develop chronic kidney disease, such as diabetes mellitus, coronary heart disease, and hepatitis C, are common among patient with liver diseases.[113,114] It is therefore important to identify which patients with liver disease truly have renal impairment.[60]

#### Summary of the selected articles

Two studies were included. The mean bias ranged from −10 ml/min/1.73m^2^ with the MDRD-6 formula in the study of Mindikoglu et al. to 19 ml/min/1.73m^2^ with the MDRD-4 formula in the study of Rognant et al..[114,115] The corresponding imprecision expressed as SD were 14 and 25 ml/min/1.73m^2^, respectively.[113,115] Both bias and imprecision were very wide for patients with a mGFR < 60 ml/min/1.73m^2^. Both over- and underestimation were reported, which resulted in an accuracy expressed as P_30_ of 40 to 62%.[113,115] The number of patients in the separate studies were below 100, but the measurements of the GFR were adequately performed.

#### Interpretation and conclusion

The imprecision of the MDRD formula in the studies was very wide. Cholongitas et al. already reported that the MDRD overestimates the GFR to a great extent in a review in 2007.[112] The two more recently published articles confirm this conclusion. Remarkably, the study in which the MDRD-6 formula was used, reported an underestimation of the mGFR.[115] Despite the fact that only two studies met our selection criteria and per study less than 100 patients were included, the degree of over- and underestimation and precision is too large to justify the use of the MDRD formula in patients with liver cirrhosis and moderate to severe renal impairment.

### Human immunodeficiency virus (HIV)

#### Background

Individuals with HIV infection have an increased risk of kidney disease.[116] HIV infection may result in HIV-associated nephropathy, immune complex kidney disease and ARF.[117,118] Moreover, progression to end-stage kidney disease, which may require hemodialysis, is common.[119,120,121] These conditions are associated with progression to acquired immune deficiency syndrome (AIDS) and death.[118,119] In addition, HIV itself may influence other risk factors for kidney disease, such as lipid levels, insulin resistance and microalbuminuria.[122] Aging, comorbidities and the use of nephrotoxic antiretroviral drugs might lead to a higher risk for developing impaired renal function.[122,123,124] In addition, the use of renally excreted drugs is prevalent, therefore accurate estimation of renal function is an important component of personalized HIV care.[117,125]

#### Summary of the selected articles

We selected two subanalysis including only 45 patients with HIV and mGFR < 60 ml/min/1.73m^2^. The imprecision reported was very wide, namely an IQR of 20 ml/min/1.73m^2^ in the study of Inker et al. and mean relative bias of 61% in the study of Gagneux et al..[126,127] This resulted in an accuracy, expressed as P_30,_ of 67% and 22%, respectively.[126,127] The measurement of GFR with the gold standard iohexol was adequately performed in the study of Inker et al. and reasonably performed in the study of Gagneux et al.[126,127]

#### Interpretation and conclusion

We were not able to draw conclusions about the validity of the MDRD formula in patients with HIV and moderate to severe renal impairment, because of the small number of patients. The wide imprecision reported in the small subanalysis does not support the validity of the MDRD formula.

Of note, in our previously published review about the validity of the MDRD formula in HIV-infected patients we suggested that the MDRD-4 formula is as valid in HIV-positive as in HIV-negative patients.[128] The results in this review do not confirm that hypothesis for patients with moderate to severe renal impairment.

## Discussion

To our knowledge this is the first systematic review, which evaluates the validity of the MDRD formula in a range of specific patient populations with moderate to severe renal impairment in a more quantitative way. We focused on studies, which compared the MDRD formula with a gold standard and which provided statistical outcome information about the degree of deviation from the true GFR. Our selection criteria were thus more stringent than previously published reviews.[3,11,129]

This review showed that the validity of the MDRD formula has not yet been tested properly in patients with cardiovascular diseases and chronic respiratory diseases. In obese patients, patients with cancer and HIV the validity of the MDRD formula has been poorly tested. The number of studies and/or the number of patients included were very low and/or the measurement of GFR was not performed adequately. Therefore the validity of the MDRD formula in these patient populations remains unclear. For patients with diabetes mellitus and liver cirrhosis, hospitalized patients on the internal medicine and nephrology ward and elderly with moderate to severe renal impairment we concluded that the MDRD formula is not valid. A summary is given in Table 3.

**Table 3:** Validity of the MDRD formula in different patient populations.

Patient population	Validity of the MDRD formula
Elderly patients	Not valid
Hospitalized patients*	Not valid
Obese patients	Unclear
Cardiovascular diseases	Not tested
Cancer	Unclear
Chronic respiratory diseases	Not tested
Diabetes mellitus	Not valid
Liver cirrhosis	Not valid
Human immunodeficiency virus	Unclear

* The MDRD formula is not valid in patients on the internal medicine and nephrology ward. For other hospitalized patients it is not tested.

Overall, we may conclude that the application of the MDRD formula in clinical practice is not supported by available research evidence for a range of specific patient populations. The application of the MDRD formula in drug dosing may become even more difficult with the knowledge that most of these chronic diseases are present in various combinations in the individual patient, especially in elderly.[130] The variability of the eGFR in daily practice might thus be larger.

At the time this research was conducted the Chronic Kidney Disease Epidemiology Collaboration (CKD-EPI) formulas were developed. These formulas are based on serum creatinine value, cystatine value and a combination of both.[131,132] Overall, the CKD-EPI formula performs better than the MDRD-4 formula. However, the differences in the GFR range < 60 ml/min/1.73m^2^ are small and not clinically relevant.[17,133] In Australia, France and a few large laboratories in the United States, the eGFR is already calculated with the CKD-EPI formula.[134] Although the CKD-EPI formula may replace the MDRD formula, we still think that our review is of great interest. First, the limitations of the MDRD formula are due to the variable serum creatinine level. This variable still exists in the CKD-EPI formula. Second, this review shows the importance of validating a formula in specific patient populations. Especially, populations who are at risk of having impaired renal function.

This review is not without limitations. First, we excluded studies which did not fulfill our criteria concerning statistical analysis. A frequently used method to compare different formulas to estimate the GFR is the correlation coefficient, which has major limitations when used for this purpose.[117,121] The most informative method to assess diagnostic tests is the Bland Altman plot, as this identifies the direction and the magnitude of the bias.[67,135,136] Secondly, the gold standards used in the presented studies were diverse. The gold standard in the development of the MDRD formula was ^125^I-iothalamate.[15]. Use of other filtration markers may introduce a systematic bias, mostly an overestimation.[4,17,44] We did not consider this variable in the interpretation of the included studies. This would have been difficult because the measurement of the GFR was often not well described and/or not adequately performed. Another limitation is the lack of taking into account the differences between the MDRD en re-expressed MDRD formulas, in other words, between IDMS calibrated creatinine measurements and uncalibrated creatinine measurements. In addition, the use of an enzymatic method or the Jaffe method also introduces variable variations in serum creatinine levels.[2,137] We did not consider such additional variables in the interpretations of the selected studies. Instead, we choose to focus on the variables explained in the method section, which in our opinion have the greatest influence on the eGFR(MDRD). Finally, we did not discuss all different patient populations. Examples of patient characteristics which may also affect the validity of the MDRD formula, but which were not reviewed here, are pregnancy and ethnicity.[138,139,140,141]

## Conclusion

In summary, the use of the MDRD formula in different specific patient populations for the fine-tuning of drug therapy management is not without limitations. There is no hard evidence that the MDRD formula is valid in patients with several chronic diseases combined with renal impairment. Clinical judgment remains necessary. Instead of searching for the ideal formula for estimating GFR, we should search for practical approaches to optimize the pharmacotherapy in patients with renal impairment.

## Supporting Information

S1 PRISMA Checklist (PDF)

S1 File Search terms. (DOCX)

## References

[1] Zhang L, Xu N, Xiao S, Arya V, Zhao P, et al (2012) Regulatory perspectives on designing pharmacokinetic studies and optimizing labeling recommendations for patients with chronic kidney disease. J Clin Pharmacol 52: 79S–90S. 10.1177/0091270011415410 22232757

[2] Prigent A (2008) Monitoring renal function and limitations of renal function tests. Semin Nucl Med 38: 32–46. 18096462 10.1053/j.semnuclmed.2007.09.003

[3] Stevens LA, Coresh J, Greene T, Levey AS (2006) Assessing kidney function—measured and estimated glomerular filtration rate. N Engl J Med 354: 2473–2483. 16760447 10.1056/NEJMra054415

[4] Stevens LA, Levey AS (2005) Measurement of kidney function. Med Clin North Am 89: 457–473. 15755462 10.1016/j.mcna.2004.11.009

[5] Anonymous (2002) K/DOQI clinical practice guidelines for chronic kidney disease: evaluation, classification, and stratification. Am J Kidney Dis 39: S1–S266. 11904577

[6] Stevens LA, Nolin TD, Richardson MM, Feldman HI, Lewis JB, et al (2009) Comparison of drug dosing recommendations based on measured GFR and kidney function estimating equations. Am J Kidney Dis 54: 33–42. 10.1053/j.ajkd.2009.03.008 19446939 PMC2756662

[7] Pai MP (2010) Estimating the glomerular filtration rate in obese adult patients for drug dosing. Adv Chronic Kidney Dis 17: e53–e62. 10.1053/j.ackd.2010.05.010 20727504

[8] Verhave JC, Wetzels JF, Bakker SJ, Gansevoort RT (2007) Schatting van de nierfunctie met behulp van formules [estimating renal function with formulas]. Ned Tijdschr Geneeskd 151: 1002–1004. 17508682

[9] Hellden A, Bergman U, von Euler M, Hentschke M, Odar-Cederlof I, et al (2009) Adverse drug reactions and impaired renal function in elderly patients admitted to the emergency department: a retrospective study. Drugs Aging 26: 595–606. 10.2165/11315790-000000000-00000 19655826

[10] Leendertse AJ, van Dijk EA, De Smet PA, Egberts TC, van den Bemt PM (2012) Contribution of renal impairment to potentially preventable medication-related hospital admissions. Ann Pharmacother 46: 625–633. 10.1345/aph.1Q633 22570433

[11] Helou R (2010) Should we continue to use the Cockcroft-Gault formula? Nephron Clin Pract 116: c172–c185. 10.1159/000317197 20606477

[12] Botev R, Mallie JP, Couchoud C, Schuck O, Fauvel JP, et al (2009) Estimating glomerular filtration rate: Cockcroft-Gault and Modification of Diet in Renal Disease formulas compared to renal inulin clearance. Clin J Am Soc Nephrol 4: 899–906. 10.2215/CJN.05371008 19406960 PMC2676189

[13] Cockcroft DW, Gault MH (1976) Prediction of creatinine clearance from serum creatinine. Nephron 16: 31–41. 1244564 10.1159/000180580

[14] Coresh J, Auguste P (2008) Reliability of GFR formulas based on serum creatinine, with special reference to the MDRD Study equation. Scand J Clin Lab Invest Suppl 241: 30–38. 10.1080/00365510802141140 18569962

[15] Levey AS, Bosch JP, Lewis JB, Greene T, Rogers N, et al (1999) A more accurate method to estimate glomerular filtration rate from serum creatinine: a new prediction equation. Modification of Diet in Renal Disease Study Group. Ann Intern Med 130: 461–470. 10075613 10.7326/0003-4819-130-6-199903160-00002

[16] Levey AS, Green T, Kusek JW, Beck GJ, MDRD Study Group (2000) A simplified equation to predict glomerular filtration rate from serum creatinine [abstract] 2000. J Am Soc Nephrol 11: 155A (A0828).

[17] Earley A, Miskulin D, Lamb EJ, Levey AS, Uhlig K (2012) Estimating equations for glomerular filtration rate in the era of creatinine standardization: a systematic review. Ann Intern Med 156: 785–795, W-270, W-271, W-272, W-273, W-274, W-275, W-276, W-277, W-278. 10.7326/0003-4819-156-6-201203200-00391 22312131

[18] Levey AS, Coresh J, Greene T, Marsh J, Stevens LA, et al (2007) Expressing the Modification of Diet in Renal Disease Study equation for estimating glomerular filtration rate with standardized serum creatinine values. Clin Chem 53: 766–772. 17332152 10.1373/clinchem.2006.077180

[19] Mathew TH, Johnson DW, Jones GR (2007) Chronic kidney disease and automatic reporting of estimated glomerular filtration rate: revised recommendations. Med J Aust 187: 459–463. 17937643 10.5694/j.1326-5377.2007.tb01357.x

[20] Stevens LA, Levey AS (2009) Use of the MDRD study equation to estimate kidney function for drug dosing. Clin Pharmacol Ther 86: 465–467. 10.1038/clpt.2009.124 19844220

[21] Levey AS, Coresh J, Greene T, Stevens LA, Zhang YL, et al (2006) Using standardized serum creatinine values in the modification of diet in renal disease study equation for estimating glomerular filtration rate. Ann Intern Med 145: 247–254. 16908915 10.7326/0003-4819-145-4-200608150-00004

[22] Myers GL, Miller WG, Coresh J, Fleming J, Greenberg N, et al (2006) Recommendations for improving serum creatinine measurement: a report from the Laboratory Working Group of the National Kidney Disease Education Program. Clin Chem 52: 5–18. 16332993 10.1373/clinchem.2005.0525144

[23] Spruill WJ, Wade WE, Cobb HH III (2009) Continuing the use of the Cockcroft-Gault equation for drug dosing in patients with impaired renal function. Clin Pharmacol Ther 86: 468–470. 10.1038/clpt.2009.187 19844221

[24] Frequently asked questions about GFR estimates. Available at: http://www.kidney.org/professionals/kls/pdf/12-10-4004_KBB_FAQs_AboutGFR-1.pdf: National Kidney Foundation. Accessed 4 December 2012.

[25] Joosten H, Drion I, Boogerd KJ, van der Pijl EV, Slingerland RJ, et al (2013) Optimising drug prescribing and dispensing in subjects at risk for drug errors due to renal impairment: improving drug safety in primary healthcare by low eGFR alerts. BMJ Open 3. 10.1136/bmjopen-2012-002068 PMC3563134 23355668

[26] Perrone RD, Madias NE, Levey AS (1992) Serum creatinine as an index of renal function: new insights into old concepts. Clin Chem 38: 1933–1953. 1394976

[27] Stevens LA, Levey AS (2009) Measured GFR as a confirmatory test for estimated GFR. J Am Soc Nephrol 20: 2305–2313. 10.1681/ASN.2009020171 19833901

[28] van Deventer HE, Paiker JE, Katz IJ, George JA (2011) A comparison of cystatin C- and creatinine-based prediction equations for the estimation of glomerular filtration rate in black South Africans. Nephrol Dial Transplant 26: 1553–1558. 10.1093/ndt/gfq621 20961892 PMC3108353

[29] Beddhu S, Samore MH, Roberts MS, Stoddard GJ, Pappas LM, et al (2003) Creatinine production, nutrition, and glomerular filtration rate estimation. J Am Soc Nephrol 14: 1000–1005. 12660334 10.1097/01.asn.0000057856.88335.dd

[30] Levey AS (1993) Assessing the effectiveness of therapy to prevent the progression of renal disease. Am J Kidney Dis 22: 207–214. 8322785 10.1016/s0272-6386(12)70188-9

[31] Levey AS, Stevens LA, Hostetter T (2006) Automatic reporting of estimated glomerular filtration rate—just what the doctor ordered. Clin Chem 52: 2188–2193. 17068166 10.1373/clinchem.2006.078733

[32] Andreev E, Koopman M, Arisz L (1999) A rise in plasma creatinine that is not a sign of renal failure: which drugs can be responsible? J Intern Med 246: 247–252. 10475992 10.1046/j.1365-2796.1999.00515.x

[33] Cocchetto DM, Tschanz C, Bjornsson TD (1983) Decreased rate of creatinine production in patients with hepatic disease: implications for estimation of creatinine clearance. Ther Drug Monit 5: 161–168. 6879639 10.1097/00007691-198306000-00002

[34] Fossati P, Ponti M, Passoni G, Tarenghi G, Melzi d'Eril GV, et al (1994) A step forward in enzymatic measurement of creatinine. Clin Chem 40: 130–137. 8287520

[35] Peake M, Whiting M (2006) Measurement of serum creatinine—current status and future goals. Clin Biochem Rev 27: 173–184. 17581641 PMC1784008

[36] Ducharme MP, Smythe M, Strohs G (1993) Drug-induced alterations in serum creatinine concentrations. Ann Pharmacother 27: 622–633. 8347916 10.1177/106002809302700518

[37] Quon H, Grossman CE, King RL, Putt M, Donaldson K, et al (2010) Interference with the Jaffe method for creatinine following 5-aminolevulinic acid administration. Photodiagnosis Photodyn Ther 7: 268–274. 10.1016/j.pdpdt.2010.07.008 21112550 PMC3598580

[38] Murphy JL, Hurt TL, Griswold WR, Peterson BM, Rodarte A, et al (1989) Interference with creatinine concentration measurement by high dose furosemide infusion. Crit Care Med 17: 889–890. 2766761 10.1097/00003246-198909000-00009

[39] Swain RR, Briggs SL (1977) Positive interference with the Jaffe reaction by cephalosporin antibiotics. Clin Chem 23: 1340–1342. 872385

[40] Miller WG, Myers GL, Ashwood ER, Killeen AA, Wang E, et al (2005) Creatinine measurement: state of the art in accuracy and interlaboratory harmonization. Arch Pathol Lab Med 129: 297–304. 15737021 10.5858/2005-129-297-CMSOTA

[41] Myre SA, McCann J, First MR, Cluxton RJ Jr. (1987) Effect of trimethoprim on serum creatinine in healthy and chronic renal failure volunteers. Ther Drug Monit 9: 161–165. 3617154 10.1097/00007691-198706000-00006

[42] Kemperman FA, Silberbusch J, Slaats EH, van Zanten AP, Weber JA, et al (1999) Glomerular filtration rate estimation from plasma creatinine after inhibition of tubular secretion: relevance of the creatinine assay. Nephrol Dial Transplant 14: 1247–1251. 10344370 10.1093/ndt/14.5.1247

[43] Kabat-Koperska J, Motyl W, Domanski L, Safranow K, Golembiewska E, et al (2004) Methods of GFR determination—creatinine clearance after cimetidine administration in clinical practice. Acta Med Austriaca 31: 51–55. 15359983

[44] Hallan S, Asberg A, Lindberg M, Johnsen H (2004) Validation of the Modification of Diet in Renal Disease formula for estimating GFR with special emphasis on calibration of the serum creatinine assay. Am J Kidney Dis 44: 84–93. 15211442 10.1053/j.ajkd.2004.03.027

[45] Bjornsson TD (1979) Use of serum creatinine concentrations to determine renal function. Clin Pharmacokinet 4: 200–222. 383355 10.2165/00003088-197904030-00003

[46] Coresh J, Stevens LA (2006) Kidney function estimating equations: where do we stand? Curr Opin Nephrol Hypertens 15: 276–284. 16609295 10.1097/01.mnh.0000222695.84464.61

[47] Drenth-van Maanen AC, Jansen PA, Proost JH, Egberts TC, van Zuilen AD, et al (2013) Renal function assessment in older adults. Br J Clin Pharmacol 76: 616–623. 10.1111/bcp.12199 23802656 PMC3791984

[48] Roberts GW, Ibsen PM, Schioler CT (2009) Modified diet in renal disease method overestimates renal function in selected elderly patients. Age Ageing 38: 698–703. 10.1093/ageing/afp168 19767628

[49] Van den Noortgate NJ, Janssens WH, Delanghe JR, Afschrift MB, Lameire NH (2002) Serum cystatin C concentration compared with other markers of glomerular filtration rate in the old old. J Am Geriatr Soc 50: 1278–1282. 12133025 10.1046/j.1532-5415.2002.50317.x

[50] Martin JH, Fay MF, Udy A, Roberts J, Kirkpatrick C, et al. (2010) Pitfalls of using estimations of glomerular filtration rate in an intensive care population. Intern Med J. 10.1111/j.1445-5994.2009.02160.x 21762334

[51] Pedone C, Corsonello A, Incalzi RA (2006) Estimating renal function in older people: a comparison of three formulas. Age Ageing 35: 121–126. 16495291 10.1093/ageing/afj041

[52] Macunluoglu B, Gokce I, Atakan A, Demirci M, Ari E, et al (2011) A comparison of different methods for the determination of glomerular filtration rate in elderly patients with chronic renal failure. Int Urol Nephrol 43: 257–263. 10.1007/s11255-010-9846-0 20960232

[53] Lamb EJ, Webb MC, O'Riordan SE (2007) Using the modification of diet in renal disease (MDRD) and Cockcroft and Gault equations to estimate glomerular filtration rate (GFR) in older people. Age Ageing 36: 689–692. 17881417 10.1093/ageing/afm121

[54] facts on noncommunicable diseases. Available at: http://wwwwhoint/features/factfiles/noncommunicable_diseases/en/indexhtml. pp. Accessed 4 October 2012.

[55] Smilde TD, van Veldhuisen DJ, Navis G, Voors AA, Hillege HL (2006) Drawbacks and prognostic value of formulas estimating renal function in patients with chronic heart failure and systolic dysfunction. Circulation 114: 1572–1580. 17015793 10.1161/CIRCULATIONAHA.105.610642

[56] Waldum B, Westheim AS, Sandvik L, Flonaes B, Grundtvig M, et al (2010) Renal function in outpatients with chronic heart failure. J Card Fail 16: 374–380. 10.1016/j.cardfail.2010.01.001 20447572

[57] Iliadis F, Didangelos T, Ntemka A, Makedou A, Moralidis E, et al (2011) Glomerular filtration rate estimation in patients with type 2 diabetes: creatinine- or cystatin C-based equations? Diabetologia 54: 2987–2994. 10.1007/s00125-011-2307-1 21947381

[58] Sherman DS, Fish DN, Teitelbaum I (2003) Assessing renal function in cirrhotic patients: problems and pitfalls. Am J Kidney Dis 41: 269–278. 12552488 10.1053/ajkd.2003.50035

[59] Vrouenraets SM, Fux CA, Wit FW, Garcia EF, Brinkman K, et al (2012) A comparison of measured and estimated glomerular filtration rate in successfully treated HIV-patients with preserved renal function. Clin Nephrol 77: 311–320. 22445475 10.5414/cn107214

[60] MacAulay J, Thompson K, Kiberd BA, Barnes DC, Peltekian KM (2006) Serum creatinine in patients with advanced liver disease is of limited value for identification of moderate renal dysfunction: are the equations for estimating renal function better? Can J Gastroenterol 20: 521–526. 16955148 10.1155/2006/858053 PMC2659934

[61] Al-Aloul M, Jackson M, Bell G, Ledson M, Walshaw M (2007) Comparison of methods of assessment of renal function in cystic fibrosis (CF) patients. J Cyst Fibros 6: 41–47. 16807143 10.1016/j.jcf.2006.05.004

[62] Karstila K, Harmoinen AP, Lehtimaki TJ, Korpela MM, Mustonen JT, et al (2008) Measurement of the kidney function in patients with rheumatoid arthritis: plasma cystatin C versus 51Cr-EDTA clearance. Nephron Clin Pract 108: c284–c290. 10.1159/000127362 18434750

[63] Francoz C, Prie D, Abdelrazek W, Moreau R, Mandot A, et al (2010) Inaccuracies of creatinine and creatinine-based equations in candidates for liver transplantation with low creatinine: impact on the model for end-stage liver disease score. Liver Transpl 16: 1169–1177. 10.1002/lt.22128 20879015

[64] Vivier PH, Storey P, Rusinek H, Zhang JL, Yamamoto A, et al (2011) Kidney function: glomerular filtration rate measurement with MR renography in patients with cirrhosis. Radiology 259: 462–470. 10.1148/radiol.11101338 21386050 PMC6939953

[65] Agarwal R (2003) Ambulatory GFR measurement with cold iothalamate in adults with chronic kidney disease. Am J Kidney Dis 41: 752–759. 12666061 10.1016/s0272-6386(03)00022-2

[66] Fehrman-Ekholm I, Seeberger A, Bjork J, Sterner G (2009) Serum cystatin C: a useful marker of kidney function in very old people. Scand J Clin Lab Invest 69: 606–611. 10.1080/00365510903015771 19517296

[67] Stevens LA, Zhang Y, Schmid CH (2008) Evaluating the performance of equations for estimating glomerular filtration rate. J Nephrol 21: 797–807. 19034863 PMC4418188

[68] Shemesh O, Golbetz H, Kriss JP, Myers BD (1985) Limitations of creatinine as a filtration marker in glomerulopathic patients. Kidney Int 28: 830–838. 2418254 10.1038/ki.1985.205

[69] Van Pottelbergh G, Van Heden L, Mathei C, Degryse J (2010) Methods to evaluate renal function in elderly patients: a systematic literature review. Age Ageing 39: 542–548. 10.1093/ageing/afq091 20716584

[70] Horio M, Yasuda Y, Takahara S, Imai E, Watanabe T, et al (2010) Comparison of a simple and a standard method for inulin renal clearance. Clin Exp Nephrol 14: 427–430. 10.1007/s10157-010-0325-9 20661617

[71] Koppe L, Klich A, Dubourg L, Ecochard R, Hadj-Aissa A (2013) Performance of creatinine-based equations compared in older patients. J Nephrol 26: 716–723. 10.5301/jn.5000297 23843047

[72] Quartarolo JM, Thoelke M, Schafers SJ (2007) Reporting of estimated glomerular filtration rate: effect on physician recognition of chronic kidney disease and prescribing practices for elderly hospitalized patients. J Hosp Med 2: 74–78. 17427247 10.1002/jhm.172

[73] Fliser D (2008) Assessment of renal function in elderly patients. Curr Opin Nephrol Hypertens 17: 604–608. 10.1097/MNH.0b013e32830f454e 18941354

[74] Lamb EJ, Webb MC, Simpson DE, Coakley AJ, Newman DJ, et al (2003) Estimation of glomerular filtration rate in older patients with chronic renal insufficiency: is the modification of diet in renal disease formula an improvement? J Am Geriatr Soc 51: 1012–1017. 12834524 10.1046/j.1365-2389.2003.51330.x

[75] Nygaard HA, Naik M, Ruths S, Kruger K (2004) Clinically important renal impairment in various groups of old persons. Scand J Prim Health Care 22: 152–156. 15370791 10.1080/02813430410006468

[76] Bevc S, Hojs R, Ekart R, Gorenjak M, Puklavec L (2011) Simple cystatin C formula compared to sophisticated CKD-EPI formulas for estimation of glomerular filtration rate in the elderly. Ther Apher Dial 15: 261–268. 10.1111/j.1744-9987.2011.00948.x 21624073

[77] Kilbride HS, Stevens PE, Eaglestone G, Knight S, Carter JL, et al. (2012) Accuracy of the MDRD (Modification of Diet in Renal Disease) Study and CKD-EPI (CKD Epidemiology Collaboration) Equations for Estimation of GFR in the Elderly. Am J Kidney Dis. 10.1053/j.ajkd.2012.06.016 22889713

[78] Froissart M, Rossert J, Jacquot C, Paillard M, Houillier P (2005) Predictive performance of the modification of diet in renal disease and Cockcroft-Gault equations for estimating renal function. J Am Soc Nephrol 16: 763–773. 15659562 10.1681/ASN.2004070549

[79] Stevens LA, Coresh J, Feldman HI, Greene T, Lash JP, et al (2007) Evaluation of the modification of diet in renal disease study equation in a large diverse population. J Am Soc Nephrol 18: 2749–2757. 17855641 10.1681/ASN.2007020199

[80] Evans M, van Stralen KJ, Schon S, Prutz KG, Stendahl M, et al (2013) Glomerular filtration rate-estimating equations for patients with advanced chronic kidney disease. Nephrol Dial Transplant 28: 2518–2526. 10.1093/ndt/gft226 23904399

[81] Lipcsey M, Furebring M, Rubertsson S, Larsson A (2011) Significant differences when using creatinine, modification of diet in renal disease, or cystatin C for estimating glomerular filtration rate in ICU patients. Ups J Med Sci 116: 39–46. 10.3109/03009734.2010.526724 21067456 PMC3039759

[82] Bouchard J, Macedo E, Soroko S, Chertow GM, Himmelfarb J, et al (2010) Comparison of methods for estimating glomerular filtration rate in critically ill patients with acute kidney injury. Nephrol Dial Transplant 25: 102–107. 10.1093/ndt/gfp392 19679558 PMC2910324

[83] Chertow GM, Burdick E, Honour M, Bonventre JV, Bates DW (2005) Acute kidney injury, mortality, length of stay, and costs in hospitalized patients. J Am Soc Nephrol 16: 3365–3370. 16177006 10.1681/ASN.2004090740

[84] Hoste EA, Damen J, Vanholder RC, Lameire NH, Delanghe JR, et al (2005) Assessment of renal function in recently admitted critically ill patients with normal serum creatinine. Nephrol Dial Transplant 20: 747–753. 15701668 10.1093/ndt/gfh707

[85] Poggio ED, Nef PC, Wang X, Greene T, Van LF, et al (2005) Performance of the Cockcroft-Gault and modification of diet in renal disease equations in estimating GFR in ill hospitalized patients. Am J Kidney Dis 46: 242–252. 16112042 10.1053/j.ajkd.2005.04.023

[86] Schuck O, Teplan V, Mareckova O, Skibova J, Stollova M (2005) Estimation of glomerular filtration rate based on the modification of diet in renal disease equation in patients with chronic renal failure. Kidney Blood Press Res 28: 63–67. 15640609 10.1159/000083238

[87] Hanley MJ, Abernethy DR, Greenblatt DJ (2010) Effect of obesity on the pharmacokinetics of drugs in humans. Clin Pharmacokinet 49: 71–87. 10.2165/11318100-000000000-00000 20067334

[88] de Boer IH, Katz R, Fried LF, Ix JH, Luchsinger J, et al (2009) Obesity and change in estimated GFR among older adults. Am J Kidney Dis 54: 1043–1051. 10.1053/j.ajkd.2009.07.018 19782454 PMC2787647

[89] Bouquegneau A, Vidal-Petiot E, Vrtovsnik F, Cavalier E, Rorive M, et al (2013) Modification of Diet in Renal Disease versus Chronic Kidney Disease Epidemiology Collaboration equation to estimate glomerular filtration rate in obese patients. Nephrol Dial Transplant 28 Suppl 4: iv122–130. 10.1093/ndt/gft329 24026245

[90] Luciani R, Lazzarino AI, Capuano F, Benedetto U, Goracci M, et al (2010) Preoperative creatinine clearance as a predictor of short-term outcomes after cardiac surgery: a cohort study for the comparison between the Cockroft-Gault and modification of diet in renal disease formulae. J Cardiovasc Med (Hagerstown) 11: 271–275. 10.2459/JCM.0b013e328336b558 20072000

[91] Ruilope LM, Zanchetti A, Julius S, McInnes GT, Segura J, et al (2007) Prediction of cardiovascular outcome by estimated glomerular filtration rate and estimated creatinine clearance in the high-risk hypertension population of the VALUE trial. J Hypertens 25: 1473–1479. 17563571 10.1097/HJH.0b013e328133246c

[92] Matsushita K, Selvin E, Bash LD, Astor BC, Coresh J (2010) Risk implications of the new CKD Epidemiology Collaboration (CKD-EPI) equation compared with the MDRD Study equation for estimated GFR: the Atherosclerosis Risk in Communities (ARIC) Study. Am J Kidney Dis 55: 648–659. 10.1053/j.ajkd.2009.12.016 20189275 PMC2858455

[93] Perticone F, Maio R, Ruberto C, Cassano S, Tripepi G, et al (2008) Kidney function and risk factors for left ventricular hypertrophy in untreated uncomplicated essential hypertension. Am J Kidney Dis 52: 74–84. 10.1053/j.ajkd.2008.02.302 18423813

[94] Del Fabbro P, Luthi JC, Carrera E, Michel P, Burnier M, et al (2010) Anemia and chronic kidney disease are potential risk factors for mortality in stroke patients: a historic cohort study. BMC Nephrol 11: 27 10.1186/1471-2369-11-27 20950484 PMC2973927

[95] O'Meara E, Chong KS, Gardner RS, Jardine AG, Neilly JB, et al (2006) The Modification of Diet in Renal Disease (MDRD) equations provide valid estimations of glomerular filtration rates in patients with advanced heart failure. Eur J Heart Fail 8: 63–67. 16084759 10.1016/j.ejheart.2005.04.013

[96] Abdo AS, Basu A, Geraci SA (2011) Managing chronic heart failure patient in chronic kidney disease. Am J Med 124: 26–28. 10.1016/j.amjmed.2010.05.030 20932501

[97] Geerts AF, De Koning FH, De Smet PA, Van Solinge WW, Egberts TC (2009) Laboratory tests in the clinical risk management of potential drug-drug interactions: a cross-sectional study using drug-dispensing data from 100 Dutch community pharmacies. Drug Saf 32: 1189–1197. 10.2165/11316700-000000000-00000 19916585

[98] Fawaz A, Badr KF (2006) Measuring filtration function in clinical practice. Curr Opin Nephrol Hypertens 15: 643–647. 17053481 10.1097/01.mnh.0000247504.94785.98

[99] Aapro M, Launay-Vacher V (2012) Importance of monitoring renal function in patients with cancer. Cancer Treat Rev 38: 235–240. 10.1016/j.ctrv.2011.05.001 21605937

[100] Launay-Vacher V, Oudard S, Janus N, Gligorov J, Pourrat X, et al (2007) Prevalence of Renal Insufficiency in cancer patients and implications for anticancer drug management: the renal insufficiency and anticancer medications (IRMA) study. Cancer 110: 1376–1384. 17634949 10.1002/cncr.22904

[101] Janus N, Launay-Vacher V, Byloos E, Machiels JP, Duck L, et al (2010) Cancer and renal insufficiency results of the BIRMA study. Br J Cancer 103: 1815–1821. 10.1038/sj.bjc.6605979 21063408 PMC3008606

[102] Janus N, Thariat J, Boulanger H, Deray G, Launay-Vacher V (2010) Proposal for dosage adjustment and timing of chemotherapy in hemodialyzed patients. Ann Oncol 21: 1395–1403. 10.1093/annonc/mdp598 20118214

[103] Marx GM, Blake GM, Galani E, Steer CB, Harper SE, et al (2004) Evaluation of the Cockroft-Gault, Jelliffe and Wright formulae in estimating renal function in elderly cancer patients. Ann Oncol 15: 291–295. 14760124 10.1093/annonc/mdh079

[104] Bolke E, Schieren G, Gripp S, Steinbach G, Peiper M, et al (2011) Cystatin C—a fast and reliable biomarker for glomerular filtration rate in head and neck cancer patients. Strahlenther Onkol 187: 191–201. 10.1007/s00066-010-2203-5 21359659

[105] Faluyi OO, Masinghe SP, Hayward RL, Clive S (2011) Accuracy of GFR estimation by the Cockroft and Gault, MDRD, and Wright equations in Oncology patients with renal impairment. Med Oncol. 10.1007/s12032-011-9824-0 21286862

[106] Ainsworth NL, Marshall A, Hatcher H, Whitehead L, Whitfield GA, et al (2012) Evaluation of glomerular filtration rate estimation by Cockcroft-Gault, Jelliffe, Wright and Modification of Diet in Renal Disease (MDRD) formulae in oncology patients. Ann Oncol 23: 1845–1853. 10.1093/annonc/mdr539 22104575

[107] Incalzi RA, Corsonello A, Pedone C, Battaglia S, Paglino G, et al (2010) Chronic renal failure: a neglected comorbidity of COPD. Chest 137: 831–837. 10.1378/chest.09-1710 19903974

[108] Gjerde B, Bakke PS, Ueland T, Hardie JA, Eagan TM (2012) The prevalence of undiagnosed renal failure in a cohort of COPD patients in western Norway. Respir Med 106: 361–366. 10.1016/j.rmed.2011.10.004 22129490

[109] Rigalleau V, Beauvieux MC, Gonzalez C, Raffaitin C, Lasseur C, et al (2011) Estimation of renal function in patients with diabetes. Diabetes Metab 37: 359–366. 10.1016/j.diabet.2011.05.002 21680218

[110] Rigalleau V, Lasseur C, Perlemoine C, Barthe N, Raffaitin C, et al (2005) Estimation of glomerular filtration rate in diabetic subjects: Cockcroft formula or modification of Diet in Renal Disease study equation? Diabetes Care 28: 838–843. 15793182 10.2337/diacare.28.4.838

[111] Chudleigh RA, Ollerton RL, Dunseath G, Peter R, Harvey JN, et al (2009) Use of cystatin C-based estimations of glomerular filtration rate in patients with type 2 diabetes. Diabetologia 52: 1274–1278. 10.1007/s00125-009-1379-7 19430759

[112] Cholongitas E, Shusang V, Marelli L, Nair D, Thomas M, et al (2007) Review article: renal function assessment in cirrhosis—difficulties and alternative measurements. Aliment Pharmacol Ther 26: 969–978. 17877504 10.1111/j.1365-2036.2007.03443.x

[113] Gerhardt T, Poge U, Stoffel-Wagner B, Palmedo H, Sauerbruch T, et al (2011) Creatinine-based glomerular filtration rate estimation in patients with liver disease: the new Chronic Kidney Disease Epidemiology Collaboration equation is not better. Eur J Gastroenterol Hepatol 23: 969–973. 10.1097/MEG.0b013e32834991f1 21897265

[114] Rognant N, Bacchetta J, Dubourg L, Ahmed SN, Radenne S, et al (2010) What is the best alternative to inulin clearance to estimate GFR in patients with decompensated alcoholic cirrhosis? Nephrol Dial Transplant 25: 3569–3575. 10.1093/ndt/gfq248 20466685

[115] Mindikoglu AL, Dowling TC, Weir MR, Seliger SL, Christenson RH, et al (2014) Performance of chronic kidney disease epidemiology collaboration creatinine-cystatin C equation for estimating kidney function in cirrhosis. Hepatology 59: 1532–1542. 10.1002/hep.26556 23744636 PMC3883887

[116] Jones CY, Jones CA, Wilson IB, Knox TA, Levey AS, et al (2008) Cystatin C and creatinine in an HIV cohort: the nutrition for healthy living study. Am J Kidney Dis 51: 914–924. 10.1053/j.ajkd.2008.01.027 18455851 PMC4430838

[117] Ravasi G, Lauriola M, Tinelli C, Brandolini M, Uglietti A, et al (2009) Comparison of glomerular filtration rate estimates vs. 24-h creatinine clearance in HIV-positive patients. HIV Med 10: 219–228. 10.1111/j.1468-1293.2008.00673.x 19187174

[118] Mocroft A, Kirk O, Reiss P, De Wit S, Sedlacek D, et al (2010) Estimated glomerular filtration rate, chronic kidney disease and antiretroviral drug use in HIV-positive patients. AIDS 24: 1667–1678. 10.1097/QAD.0b013e328339fe53 20523203

[119] Gupta SK, Eustace JA, Winston JA, Boydstun II, Ahuja TS, et al (2005) Guidelines for the management of chronic kidney disease in HIV-infected patients: recommendations of the HIV Medicine Association of the Infectious Diseases Society of America. Clin Infect Dis 40: 1559–1585. 15889353 10.1086/430257

[120] Beringer PM, Owens H, Nguyen A, Mordwinkin N, Louie S, et al (2010) Estimation of glomerular filtration rate by using serum cystatin C and serum creatinine concentrations in patients with human immunodeficiency virus. Pharmacotherapy 30: 1004–1010. 10.1592/phco.30.10.1004 20874037

[121] Bonjoch A, Bayes B, Riba J, Puig J, Estany C, et al (2010) Validation of estimated renal function measurements compared with the isotopic glomerular filtration rate in an HIV-infected cohort. Antiviral Res 88: 347–354. 10.1016/j.antiviral.2010.09.015 20887753

[122] Odden MC, Scherzer R, Bacchetti P, Szczech LA, Sidney S, et al (2007) Cystatin C level as a marker of kidney function in human immunodeficiency virus infection: the FRAM study. Arch Intern Med 167: 2213–2219. 17998494 10.1001/archinte.167.20.2213 PMC3189482

[123] Mauss S, Berger F, Kuschak D, Henke J, Hegener P, et al (2008) Cystatin C as a marker of renal function is affected by HIV replication leading to an underestimation of kidney function in HIV patients. Antivir Ther 13: 1091–1095. 19195336

[124] Praditpornsilpa K, Avihingsanon A, Chaiwatanarat T, Chaiyahong P, Wongsabut J, et al (2012) Comparisons between validated estimated glomerular filtration rate equations and isotopic glomerular filtration rate in HIV patients. AIDS 26: 1781–1788. 10.1097/QAD.0b013e328356480d 22713478 PMC3782632

[125] Izzedine H, Launay-Vacher V, Deray G (2004) Antiretroviral drugs and the kidney: dosage adjustment and renal tolerance. Curr Pharm Des 10: 4071–4079. 15579089 10.2174/1381612043382431

[126] Gagneux-Brunon A, Delanaye P, Maillard N, Fresard A, Basset T, et al (2013) Performance of creatinine and cystatin C-based glomerular filtration rate estimating equations in a European HIV-positive cohort. Aids 27: 1573–1581. 10.1097/QAD.0b013e32835fac30 23435293

[127] Inker LA, Wyatt C, Creamer R, Hellinger J, Hotta M, et al. (2012) Performance of Creatinine and Cystatin C GFR Estimating Equations in an HIV-positive population on Antiretrovirals. J Acquir Immune Defic Syndr. 10.1097/QAI.0b013e31826a6c4f PMC3598619 22842844

[128] Eppenga WL, van Luin M, Richter C, Derijks HJ, De Smet PA, et al (2014) The validity of the modification of diet in renal disease formula in HIV-infected patients: a systematic review. J Nephrol 27: 11–18. 10.1007/s40620-013-0012-5 24519861

[129] Hudson JQ, Nyman HA (2011) Use of estimated glomerular filtration rate for drug dosing in the chronic kidney disease patient. Curr Opin Nephrol Hypertens 20: 482–491. 10.1097/MNH.0b013e328348c11f 21709552

[130] Hoffman C, Rice D, Sung HY (1996) Persons with chronic conditions. Their prevalence and costs. Jama 276: 1473–1479. 8903258

[131] Inker LA, Schmid CH, Tighiouart H, Eckfeldt JH, Feldman HI, et al (2012) Estimating glomerular filtration rate from serum creatinine and cystatin C. N Engl J Med 367: 20–29. 10.1056/NEJMoa1114248 22762315 PMC4398023

[132] Levey AS, Stevens LA, Schmid CH, Zhang YL, Castro AF III, et al (2009) A new equation to estimate glomerular filtration rate. Ann Intern Med 150: 604–612. 19414839 10.7326/0003-4819-150-9-200905050-00006 PMC2763564

[133] Delanaye P, Pottel H, Botev R, Inker LA, Levey AS (2013) Con: Should we abandon the use of the MDRD equation in favour of the CKD-EPI equation? Nephrol Dial Transplant 28: 1396–1403; discussion 1403. 10.1093/ndt/gft006 23780677

[134] Levey AS, Inker LA, Coresh J (2014) GFR estimation: from physiology to public health. Am J Kidney Dis 63: 820–834. 10.1053/j.ajkd.2013.12.006 24485147 PMC4001724

[135] Bland JM, Altman DG (1986) Statistical methods for assessing agreement between two methods of clinical measurement. Lancet 1: 307–310. 2868172

[136] Hanneman SK (2008) Design, analysis, and interpretation of method-comparison studies. AACN Adv Crit Care 19: 223–234. 10.1097/01.AACN.0000318125.41512.a3 18560291 PMC2944826

[137] Verhave JC, Fesler P, Ribstein J, du Cailar G, Mimran A (2005) Estimation of renal function in subjects with normal serum creatinine levels: influence of age and body mass index. Am J Kidney Dis 46: 233–241. 16112041 10.1053/j.ajkd.2005.05.011

[138] Lee CS, Cha RH, Lim YH, Kim H, Song KH, et al (2010) Ethnic coefficients for glomerular filtration rate estimation by the Modification of Diet in Renal Disease study equations in the Korean population. J Korean Med Sci 25: 1616–1625. 10.3346/jkms.2010.25.11.1616 21060751 PMC2966999

[139] Ahmed SB, Bentley-Lewis R, Hollenberg NK, Graves SW, Seely EW (2009) A comparison of prediction equations for estimating glomerular filtration rate in pregnancy. Hypertens Pregnancy 28: 243–255. 10.1080/10641950801986720 19440935 PMC3811128

[140] Ma YC, Zuo L, Chen JH, Luo Q, Yu XQ, et al (2006) Modified glomerular filtration rate estimating equation for Chinese patients with chronic kidney disease. J Am Soc Nephrol 17: 2937–2944. 16988059 10.1681/ASN.2006040368

[141] Matsuo S, Imai E, Horio M, Yasuda Y, Tomita K, et al (2009) Revised equations for estimated GFR from serum creatinine in Japan. Am J Kidney Dis 53: 982–992. 10.1053/j.ajkd.2008.12.034 19339088

[142] de Lemos ML, Hsieh T, Hamata L, Levin A, Swenerton K, et al (2006) Evaluation of predictive formulae for glomerular filtration rate for carboplatin dosing in gynecological malignancies. Gynecol Oncol 103: 1063–1069. 16875719 10.1016/j.ygyno.2006.06.024

[143] Lopes MB, Araujo LQ, Passos MT, Nishida SK, Kirsztajn GM, et al (2013) Estimation of glomerular filtration rate from serum creatinine and cystatin C in octogenarians and nonagenarians. BMC Nephrol 14: 265 10.1186/1471-2369-14-265 24295505 PMC4219437

[144] Fontsere N, Bonal J, Navarro M, Riba J, Fraile M, et al (2006) A comparison of prediction equations for estimating glomerular filtration rate in adult patients with chronic kidney disease stages 4–5. Effect of nutritional status and age. Nephron Clin Pract 104: c160–168. 16943683 10.1159/000095476

[145] Frank M, Guarino-Gubler S, Burnier M, Maillard M, Keller F, et al (2012) Estimation of glomerular filtration rate in hospitalised patients: are we overestimating renal function? Swiss Med Wkly 142: w13708 10.4414/smw.2012.13708 23254922

[146] Valente MA, Hillege HL, Navis G, Voors AA, Dunselman PH, et al (2014) The Chronic Kidney Disease Epidemiology Collaboration equation outperforms the Modification of Diet in Renal Disease equation for estimating glomerular filtration rate in chronic systolic heart failure. Eur J Heart Fail 16: 86–94. 10.1093/eurjhf/hft128 23901055

[147] Craig AJ, Samol J, Heenan SD, Irwin AG, Britten A (2012) Overestimation of carboplatin doses is avoided by radionuclide GFR measurement. Br J Cancer 107: 1310–1316. 10.1038/bjc.2012.393 22935580 PMC3494427

[148] Rognant N, Lemoine S, Laville M, Hadj-Aissa A, Dubourg L (2011) Performance of the chronic kidney disease epidemiology collaboration equation to estimate glomerular filtration rate in diabetic patients. Diabetes Care 34: 1320–1322. 10.2337/dc11-0203 21540431 PMC3114318

[149] Fontsere N, Bonal J, Salinas I, de Arellano MR, Rios J, et al (2008) Is the new Mayo Clinic Quadratic equation useful for the estimation of glomerular filtration rate in type 2 diabetic patients? Diabetes Care 31: 2265–2267. 10.2337/dc08-0958 18835955 PMC2584175

[150] Rigalleau V, Lasseur C, Raffaitin C, Beauvieux MC, Barthe N, et al (2007) Normoalbuminuric renal-insufficient diabetic patients: a lower-risk group. Diabetes Care 30: 2034–2039. 17485574 10.2337/dc07-0140

