# Supplementary material for: Individualizing Pharmacotherapy in Patients with Renal Impairment: The Validity of the Modification of Diet in Renal Disease Formula in Specific Patient Populations with a Glomerular Filtration Rate below 60 Ml/Min. A Systematic Review
Source: PLoS One. 2015 Mar 5;10(3):e0116403. doi: 10.1371/journal.pone.0116403 (PMC4351004; doi:10.1371/journal.pone.0116403)
Supplement: S1 File — (DOCX) [file pone.0116403.s002.docx]

**File S1: Search terms**

The following search terms were used:

*Assessment of the renal function*

1. Glomerular filtration rate and creatinine

*Reliability*

1. "Predictive Value of Tests"[68] OR "Reference Values"[68] OR predictive value* OR reference value*
2. limitation OR limitations
3. pitfalls OR pitfall
4. overestimated OR underestimated OR underestimation OR overestimation OR overestimating OR underestimating
5. disturbance OR interference
6. “diagnostic errors”[MeSH Terms] OR (diagnostic AND errors)
7. "sensitivity and specificity"[MeSH Terms] OR sensitivity OR specificity
8. marker OR markers OR "Biological Markers"[68]
9. accurate OR inaccurate OR inaccuracy OR accuracy
10. performance

*Creatinine-based formulas*

1. (cockcroft AND gault) OR cockcroft-gault OR MDRD OR (modification AND diet) OR "kidney diseases"[MeSH Terms] OR (kidney AND diseases) OR renal disease

*Date of publication*

1. January 1999 – January 2014

Last date the search was performed: April 30, 2014
